# Potent Antiplasmodial Derivatives of Dextromethorphan Reveal the *Ent*-Morphinan Pharmacophore of Tazopsine-Type Alkaloids

**DOI:** 10.3390/pharmaceutics14020372

**Published:** 2022-02-07

**Authors:** Antoinette Keita, Jean-François Franetich, Maëlle Carraz, Loïse Valentin, Mallaury Bordessoules, Ludivine Baron, Pierre Bigeard, Florian Dupuy, Valentine Geay, Maurel Tefit, Véronique Sarrasin, Sylvie Michel, Catherine Lavazec, Sandrine Houzé, Dominique Mazier, Valérie Soulard, François-Hugues Porée, Romain Duval

**Affiliations:** 1 UMR 261—MERIT, IRD, Université de Paris, 4 Avenue de l’Observatoire, 75006 Paris, France; antoinettekeita22@yahoo.com (A.K.); sandrine.houze@aphp.fr (S.H.); 2 Centre d’Immunologie et des Maladies Infectieuses, INSERM, CNRS, Sorbonne Université, 75013 Paris, France; jean-francois.franetich@upmc.fr (J.-F.F.); loise.valentin@gmail.com (L.V.); mallaurybordessoulles@gmail.com (M.B.); ludivine.baron.bio@gmail.com (L.B.); pierre.bigeard71@gmail.com (P.B.); maurel.tefit@upmc.fr (M.T.); dominique.mazier@sorbonne-universite.fr (D.M.); valerie.soulard@upmc.fr (V.S.); 3 UMR 152 Pharma-Dev, IRD, UPS, Université de Toulouse, 31400 Toulouse, France; maelle.carraz@ird.fr; 4 Biopredic International, Parc d’Affaires de la Bretèche, Bldg A4, 35760 Saint-Grégoire, France; 5 Laboratoire d’Excellence GR-Ex, 75015 Paris, France; florian.dupuy@inserm.fr (F.D.); catherine.lavazec@inserm.fr (C.L.); 6 Institut Cochin, Inserm U1016, CNRS UMR 8104, Université de Paris, 75014 Paris, France; 7 UMR 8038—CiTCoM, CNRS, Université de Paris, 4 Avenue de l’Observatoire, 75006 Paris, France; valentine.geay@gmail.com (V.G.); sylvie.michel@u-paris.fr (S.M.); 8 CNR du Paludisme, AP-HP, Hôpital Bichat-Claude-Bernard, 46 Rue Henri-Huchard, 75018 Paris, France; veronique.sarrasin-hubert@aphp.fr; 9 ISCR UMR CNRS 6226, Faculté de Pharmacie, Université de Rennes 1, 2 Avenue du Pr Léon Bernard, 35000 Rennes, France

**Keywords:** malaria, *Plasmodium berghei*, *Plasmodium falciparum*, hepatic stages, blood stages, prophylaxis, tazopsine, dextromethorphan, *N*-alkylation, hit compound

## Abstract

The alkaloid tazopsine **1** was introduced in the late 2000s as a novel antiplasmodial hit compound active against *Plasmodium falciparum* hepatic stages, with the potential to develop prophylactic drugs based on this novel chemical scaffold. However, the structural determinants of tazopsine **1** bioactivity, together with the exact definition of the pharmacophore, remained elusive, impeding further development. We found that the antitussive drug dextromethorphan (DXM) **3**, although lacking the complex pattern of stereospecific functionalization of the natural hit, was harboring significant antiplasmodial activity in vitro despite suboptimal prophylactic activity in a murine model of malaria, precluding its direct repurposing against the disease. The targeted *N*-alkylation of *nor*-DXM **15** produced a small library of analogues with greatly improved activity over DXM **3** against *P. falciparum* asexual stages. Amongst these, *N*-2′-pyrrolylmethyl-*nor*-DXM **16i** showed a 2- to 36-fold superior inhibitory potency compared to tazopsine **1** and DXM **3** against *P. falciparum* liver and blood stages, with respectively 760 ± 130 nM and 2.1 ± 0.4 μM IC_50_ values, as well as liver/blood phase selectivity of 2.8. Furthermore, cpd. **16i** showed a 5- to 8-fold increase in activity relative to DXM **3** against *P. falciparum* stages I–II and V gametocytes, with 18.5 μM and 13.2 μM IC_50_ values, respectively. Cpd. **16i** can thus be considered a promising novel hit compound against malaria in the *ent*-morphinan series with putative pan cycle activity, paving the way for further therapeutic development (e.g., investigation of its prophylactic activity in vivo).

## 1. Introduction

Malaria remains the major parasitic disease in the world, responsible for 229 million cases in 87 countries in 2019, associated with >400,000 deaths [1]. Malaria is also the most important infectious cause of mortality in children between 5 and 14 years old [2], principally from the deadliest and Africa-prevalent *Plasmodium falciparum* [3]. Malaria begins with the bite of a *Plasmodium*-infected female *Anopheles* mosquito, which injects sporozoites into the skin of the mammalian host. Sporozoites readily travel into the bloodstream, traverse several liver cells, and finally home into a hepatocyte. Once inside the host cell, sporozoites actively replicate and turn into multinucleated hepatic schizonts. At the end of the hepatic phase (i.e., 2–14 days after initial invasion, depending on the *Plasmodium* species), the schizonts and host cell rupture, releasing thousands of merozoites into the blood stream. Merozoites invade red blood cells, inside which they actively replicate, leading to erythrocytic schizonts that subsequently release more merozoites, which re-infect other erythrocytes in an exponential fashion. The symptomatology of malaria is directly associated with the parasite developmental phase in the blood, which is the principal target of most antimalarial drugs [4].

Semisynthetic artemisinin derivatives (ARTDs), associated with longer half-life companion drugs in artemisinin combination therapies (ACT), exert fast curative action against parasite blood stages and remain the frontline antimalarials prescribed worldwide. However, ARTDs are threatened by the rapid spread of artemisinin-resistant *P. falciparum* strains across Southeast Asia [5] and their independent emergence recently in Africa [6,7,8], which manifest a delayed clearance phenotype under conventional drug regimens [9]. This phenomenon is a worrying continuum of the history of chemoresistance by malaria parasites, which most exclusively affects drugs targeting the parasite blood phase. Indeed, the *Plasmodium* erythrocytic phase is characterized by important parasitemia and high mutations rates, allowing the selection of drug-resistant mutants [4,10]. On the other hand, the initial asymptomatic hepatic phase of parasite development features lower parasitemia and consequently lower mutation events, thus being considered an attractive target for malaria chemoprophylaxis. Drugs possessing novel chemical scaffolds active against parasite hepatic stages, either selectively [11] or as part of a pan-active mode of action [12,13], are therefore strongly pursued in drug discovery programs.

**Figure 1:**
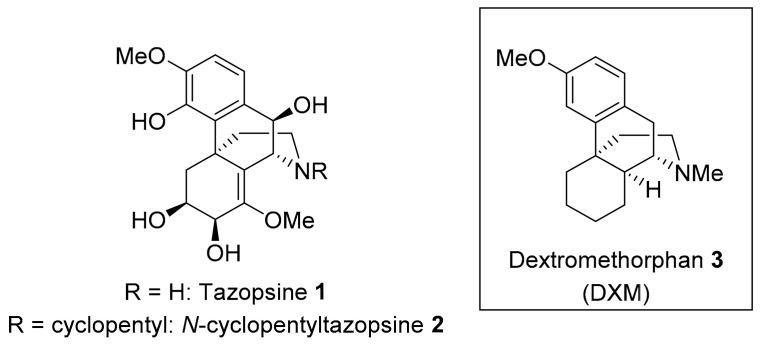
Antiplasmodial *ent*-morphinans: natural tazopsine **1**, semisynthetic hit **2**, and the prospected DXM **3**.

Tazopsine **1**, an original *ent*-morphinan alkaloid isolated from the endemic Malagasy plant *Strychnopsis thouarsii* (Figure 1), induces low micromolar inhibition of *P. falciparum* liver and blood stages in vitro, but is insufficiently prophylactic at subtoxic doses in mice infected by *P. yoelii* (70% protection at 100 mg/kg). On the other hand, the semisynthetic derivative *N*-cyclopentyltazopsine **2** is 10-fold less active, but 15-fold more selective, than **1** towards liver stages in vitro. Furthermore, **2** is also less toxic than **1**, enabling full prophylaxis at 200 mg/kg in the aforementioned malaria mouse model [14]. Despite patents filed in 2004 and 2006, these unprecedented antimalarial hits were not further investigated due to the difficult biosourcing of **1**, its complex chemical structure from a total synthesis viewpoint, limited structure–activity relationships (SAR) [14,15], as well as the absence of identified pharmacophore and biological targets in the series. However, antiplasmodial properties are shared by other *ent*-morphinan alkaloids [15,16], suggesting the existence of a common pharmacophore, irrespective of substitution and stereochemical patterns. Based on this rationale, we identified the generic antitussive drug dextromethorphan **3** (DXM **3**, 3-methoxy-17-methylmorphinan) as possibly integrating the essential functional features of *ent*-morphinan alkaloids (Figure 1), having in mind its repurposing against malaria or use as a starting material towards simplified alkaloid mimics. The present paper describes the results of these endeavors.

## 2. Experimental Section

### 2.1. Reagents, Solvents, and Equipment

Reagents and anhydrous solvents were purchased from Sigma-Aldrich and were of the highest grade available. DXM **3** hydrobromide monohydrate, sinomenine **10,** primaquine biphosphate (PQ), chloroquine biphosphate (CQ), and d-luciferin were purchased from Sigma-Aldrich. Tazopsine **1**, 10-*epi*-tazopsine **4**, sinococuline **5**, and sinoacutine **9** were isolated from the plant *Strychnopsis thouarsii* as described previously [14,15]. DX **12** and *Nor*-DXM **15** were synthesized according to literature procedures [17,18]. Column chromatography was performed using silica gel 60 (9385 Merck). Thin-layer chromatography (TLC) was performed on aluminum plates coated with silica gel 60F254 (554 Merck), visualized with UV light (254 and 366 nm), and revealed with sulfuric vanillin or phosphomolybdic acid reagents. NMR spectra (^1^H and ^13^C) were recorded on an Advance Bruker 400 MHz spectrometer or an Oxford Instruments 600 MHz spectrometer equipped with a BBI 600 MHz probe, using solvent signal as an internal standard (CDCl_3_: δ (^1^H) 7.26 and δ (^13^C) 77.16 ppm, CD_3_OD: δ (^1^H) 3.31 and δ (^13^C) 49.00 ppm). The *J* coupling constants are provided in Hertz (Hz). High-resolution mass spectra (HRMS) were recorded in the ESI mode on a LCT mass spectrometer (Waters) equipped with a TOF analyzer.

### 2.2. Synthetic Chemistry

#### 2.2.1. Chemical Derivations of Tazopsine **1** (4,6,7,10-Tetrahydroxy-8,14-didehydro-3,8- dimethoxymorphinan)

**4-*****O*****-methyl-tazopsine 6, C_19_H_25_NO_6_.** A solution of tazopsine **1** free base (20 mg, 0.057 mmol) in anhydrous MeOH (300 μL) was treated with diazomethane in excess at 0 ${}^{\circ }$C for 12 h. After removal of the solvent under reduced pressure, the residue was purified by silica gel column chromatography eluted with CH_2_Cl_2_/MeOH 0–10 *v*/*v* containing 1% of 20% aqueous NH_3_, yielding **6** (8 mg, 39%) as a white solid. ^1^H NMR (MeOD, 400 MHz): δ 6.81 (d, 1H, 8.4); 6.80 (d, 1H, *J* = 8.4); 4.45 (d, 1H, *J* = 2.1); 4.29 (d, 1H, *J* = 2.1); 4.27 (d, 1H, *J* = 2.6); 3.84 (m, 1H); 3.62 (s, 3H); 3.38 (s, 3H); 3.28 (s, 3H); 2.73 (dd, 1H, *J* = 3.2, 13.2); 2.62 (dd, 1H, *J* = 4.6, 13.9); 2.36 (ddd, 1H, *J* = 3.6, 13.9, 12.4); 2.08 (dd, 1H, *J* = 13.2, 13.2); 1.83 (dd, 1H, *J* = 3.6, 12.3); 1.79 (ddd, 1H, *J* = 4.6, 12.3, 12.4). HRMS (ESI): *m*/*z* calculated for C_19_H_26_NO_6_^+^ [M + H^+^] = 364.1760. Found = 364.1752.

**General procedure for the reductive animation of tazopsine 1 (cpds. 7a–g). ***N*-alkyl-tazopsine derivatives were obtained from tazopsine using classical reductive amination of 37% aqueous formaldehyde or pure aldehydes by NaBH_3_CN [19]. Briefly, a stirred solution of tazopsine **1** free base (34 mg, 0.097 mmol) in anhydrous MeOH (600 μL) was primed by a gentle stream of argon for 15 s. To this solution were added the aldehyde (0.107 mmol) at r. t., followed after 10 min by NaBH_3_CN (95%, 6.4 mg, 97 μmol). The mixture was stirred at r. t. under an argon atmosphere for 24 h. After removal of the solvent under reduced pressure, the residue was slowly acidified with 1 M HCl, then basified with 35% aqueous NH_3_, and evaporated under a vacuum. The residue was purified by silica gel column chromatography eluted with CH_2_Cl_2_/MeOH (0–10 *v*/*v* containing 1% of 20% aqueous NH_3_).

***N*****-methyl-tazopsine 7a, C_19_H_25_NO_6_**, 82% yield. ^1^H NMR (MeOD, 400 MHz): δ 6.90 (d, 1H, *J =* 8.4); 6.83 (d, 1H, *J =* 8.4); 4.70 (d, 1H, *J =* 2.2); 4.30 (dd, 1H, *J =* 1.3, 3.4); 4.21 (d, 1H, *J =* 2.2); 3.88 (m, 1H); 3.87 (s, 3H); 3.70 (s, 3H); 2.98 (ddd, 1H, *J =* 1.3, 4.1, 13.8); 2.51 (dd, 1H, *J =* 3.3, 12.4); 2.47 (s, 3H); 2.26 (m, 2H); 2.18 (dd, 1H, *J =* 13.8, 13.8); 1.95 (m, 2H). HRMS (ESI): *m*/*z* calculated for C_19_H_26_NO_6_^+^ [M + H^+^] = 364.1760. Found = 364.1764.

***N*****-*****n*****-propyl-tazopsine 7b, C_21_H_29_NO_6_**, 68% yield. ^1^H NMR (MeOD, 400 MHz): δ 6.99 (d, 1H, *J =* 8.4); 6.96 (d, 1H, *J =* 8.4); 4.87 (d, 1H, *J =* 1.9); 4.38 (d, 1H, *J =* 2.97); 3.92 (m, 2H); 3.88 (s, 3H); 3.78 (s, 3H); 3.09 (m, 4H); 2.27 (d, 1H, *J =* 13.4); 2.14 (dd, 1H, *J =* 4.0, 12.3); 1.79 (m, 4H); 1.04 (t, 3H, *J =* 7.3). HRMS (ESI): *m*/*z* calculated for C_21_H_30_NO_6_^+^ [M + H^+^] = 392.2073. Found = 392.2064.

***N*****-4${}^{\prime }$-hydroxybenzyl-tazopsine 7c, C_25_H_29_NO_7_**, 45% yield. ^1^H NMR (MeOD, 400 MHz): δ 7.35 (d, 2H, *J =* 8.6); 6.99 (d, 1H, *J =* 8.4); 6.92 (d, 1H, *J =* 8.4); 6.85 (d, 2H, *J =* 8.6); 4.41 (d, 1H, *J =* 3.3); 4.2 (m, 2H); 3.98 (ddd, 1H, *J =* 3.5, 3.5, 12.98); 3.88 (s, 3H); 3.78 (m, 4H); 3.05 (m, 3H); 2.73 (ddd, 1H, *J =* 4.1, 13.0, 13.0); 2.23 (m, 3H); 2.08 (dd, 1H, *J =* 2.3, 13.7). HRMS (ESI): *m*/*z* calculated for C_25_H_30_NO_7_^+^ [M + H^+^] = 456.2022. Found = 456.2018.

***N*****-4${}^{\prime }$-methoxybenzyl-tazopsine 7d, C_26_H_31_NO_7_,** 52% yield. ^1^H NMR (MeOD, 400 MHz): δ 7.47 (d, 2H, *J =* 8.6); 7.0 (d, 2H, *J =* 8.6); 6.98 (d, 1H, *J =* 8.4); 6.93 (d, 1H, *J =* 8.4); 4.88 (d, 1H, *J =* 2.3); 4.71 (d, 1H, *J =* 2.3); 4.40 (d, 1H, *J =* 2.8); 4.25 (s, 2H); 3.99 (m, 1H); 3.88 (s, 3H); 3.83 (s, 3H); 3.77 (s, 3H); 3.06 (m, 2H); 2.75 (dd, 1H, *J =* 2.8, 12.5); 2.28 (ddd, 1H, *J =* 3.2, 13.6, 13.6); 2.20 (dd, 1H, *J =* 3.9, 13.2); 2.07 (dd, 1H, *J =* 2.1, 13.2). HRMS (ESI): *m*/*z* calculated for C_26_H_32_NO_7_^+^ [M + H^+^] = 470.2179. Found = 470.2170

***N*****-3${}^{\prime }$,4${}^{\prime }$-methylenedioxybenzyl-tazopsine 7e, C_26_H_29_NO_8_**, 52% yield. ^1^H NMR (MeOD, 400 MHz): δ 7.91 (s, 1H); 7.76 (d, 1H, *J =* 8.1); 7.54 (d, 1H, *J =* 8.1); 6.93 (d, 1H, *J =* 8.4); 6.88 (d, 1H, *J =* 8.4); 5.92 (s, 2H); 4.78 (s, 2H); 4.77 (d, 1H, *J =* 2.3); 4.56 (d, 1H, *J =* 2.3); 4.33 (d, 1H, *J =* 2.8); 3.91 (m, 1H); 3.82 (s, 3H); 3.75 (s, 3H); 2.98 (dd, 1H, *J =* 3.5, 13.4); 2.65 (dd, 1H, *J =* 4.8, 14.5); 2.45 (m, 1H); 2.2 (dd, 1H, *J =* 13.4, 13.4); 2.15 (dd, 1H, *J =* 3.5, 12.5); 1.98 (ddd, 1H, *J =* 4.8, 12.5, 12.5). HRMS (ESI): *m*/*z* calculated for C_26_H_30_NO_8_^+^ [M + H^+^] = 484.1971. Found = 484.1959

***N*****-4${}^{\prime }$-chlorobenzyl-tazopsine 7f, C_25_H_28_NO_6_Cl**, 71% yield. ^1^H NMR (MeOD, 400 MHz): δ 7.48 (d, 2H, *J =* 8.3); 7.32 (d, 2H, *J =* 8.3); 6.89 (d, 1H, *J =* 8.4); 6.82 (d, 1H, *J =* 6.4); 4.69 (s, 2H); 4.65 (d, 1H, *J =* 2.1); 4.55 (d, 1H, *J =* 2.7); 4.43 (d, 1H, *J =* 2.1); 3.89 (m, 1H); 3.84 (s, 3H); 3.61 (s, 3H); 2.96 (dd, 1H, *J =* 3.2, 13.8); 2.61 (dd, 1H, *J =* 4.6, 13.9); 2.39 (ddd, 1H, *J =* 3.7, 12.7, 13.9); 2.15 (dd, 1H, *J =* 13.4, 13.8); 2.02 (dd, 1H, *J =* 3.7, 12.7); 1.94 (ddd, 1H, *J =* 4.6, 12.7, 12.7). HRMS (ESI): *m*/*z* calculated for C_25_H_29_NO_6_Cl^+^ [M + H^+^] = 474.1683. Found = 474.1678.

***N*****-4${}^{\prime }$-bromobenzyl-tazopsine 7g, C_25_H_28_NO_6_Br**, 61% yield. ^1^H NMR (MeOD, 400 MHz): δ 7.51 (d, 2H, *J =* 8.4); 7.35 (d, 2H, *J =* 8.4); 6.92 (d, 1H, *J =* 8.4); 6.88 (d, 1H, *J =* 8.4); 4.78 (d, 1H, *J =* 2.2); 4.74 (s, 2H); 4.73 (d, 1H, *J =* 2.2); 4.32 (d, 1H, *J =* 2.7); 3.93 (ddd, 1H, *J =* 2.7, 3.7, 13.3); 3.88 (s, 3H); 3.68 (s, 3H); 3.0 (dd, 1H, *J =* 2.9, 13.9); 2.66 (m, 1H); 2.46 (ddd, 1H, *J =* 3.9, 12.1, 12.1); 2.21 (dd, 1H, *J =* 13.5, 13.5); 2.05 (ddd, 1H, *J =* 4.6, 12.6, 12.6); 1.96 (dd, 1H, *J =* 3.8, 12.6). HRMS (ESI): *m*/*z* calculated for C_25_H_29_NO_6_Br^+^ [M + H^+^] = 518.1178. Found = 518.1166.

***N*****-acetyl-tazopsine 8, C_20_H_25_NO_7_.** A solution of tazopsine **1** free base (34 mg, 0.097 mmol) in anhydrous MeOH (600 μL) was stirred at r. t. before adding Ac_2_O (0.097 mmol, 9.2 μL). The mixture was stirred at r. t. under an argon atmosphere for 1 h. After removal of the solvent under reduced pressure, the residue was purified by silica gel column chromatography eluted with CH_2_Cl_2_/MeOH (0–5 *v*/*v*), yielding **8** (17 mg, 45%) as a white solid. ^1^H NMR (MeOD, 400 MHz): *first* rotamer: δ 6.88 (d, 1H, *J =* 8.5); 6.81 (d, 1H, *J =* 8.5); 5.85 (d, 1H, *J =* 2.8); 4.39 (d, 1H, *J =* 2.8); 4.26 (dd, 1H, *J =* 1.1, 2.6); 3.68 (dd, 1H, *J =* 5.8, 12.9); 3.85 (s, 3H); 3.81 (m, 1H); 3.62 (s, 3H); 3.03 (ddd, 1H, *J =* 1.1, 4.0, 10.8); 2.78 (ddd, 1H, *J =* 4.7, 12.9, 12.7); 2.23 (dd, 1H, *J =* 5.5, 10.8); 2.15 (dd, 1H, *J =* 4.7, 12.7); 2.0 (s, 3H); 1.94 (ddd, 1H, *J =* 5.8, 12.7, 12.7); *second rotamer*: δ 6.92 (d, 1H, *J* = 8.4); 6.83 (d, 1H, *J =* 8.4); 5.28 (d, 1H, *J =* 2.5); 4.49 (d, 1H, *J =* 2.5); 4.31 (dd, 1H, *J =* 1.1, 2.7); 4.12 (dd, 1H, *J =* 5.7, 13.9); 3.84 (s, 3H); 3.81 (m, 1H); 3.74 (s, 3H); 3.03 (ddd, 1H, *J =* 1.1, 4.0, 10.8); 2.31 (ddd, 1H, *J =* 4.4, 13.9, 12.8); 2.23 (dd, 1H, *J =* 5.5, 10.8); 2.21 (s, 3H); 2.13 (dd, 1H, *J =* 4.4, 12.8); 1.78 (ddd, 1H, *J =* 5.7, 12.8, 12.8). HRMS (ESI): *m*/*z* calculated for C_20_H_26_NO7^+^ [M + H^+^] = 392.1709. Found = 392.1704.

#### 2.2.2. Chemical Derivations of DXM [(9α,13α,14α)-17-Methyl-3-methoxymorphinan] **3**

**DXM 3, C_18_H_25_NO (Generation of DXM 3 free base).** A solution of NaOH (2.16 g, 5.4 mmol in 4 mL of H_2_O) was added at r. t. to a stirred suspension of dextromethorphan **3** hydrobromide monohydrate (2 g, 5.4 mmol) in 8 mL CHCl_3_, and the resulting mixture was stirred for 30 min at r. t. The layers were separated in a separatory funnel and the organic phase was retrieved, dried over MgSO_4_, and filtered. The solvent was evaporated under reduced pressure to produce **3** free base as a dense and viscous off-white oil that solidified upon standing (1.45 g, 5.3 mmol, 99% yield). The analytical data were in accordance with the literature [18].

**2-I-DXM 11, (9α,13α,14α)-2-iodo-17-methyl-3-methoxymorphinan, C_18_H_24_INO.** To a light-protected solution of DXM **3** free base (21 mg, 0.077 mmol) in MeCN (1 mL) at 0 ${}^{\circ }$C, *N*-iodosuccinimide (20 mg, 0.089 mmol) was added, followed by *p*-toluenesulfonic acid monohydrate (27 mg, 0.14 mmol). The mixture was allowed to warm to r. t. and was stirred overnight. The reaction mixture was treated with water (1.5 mL) and 1 M Na_2_S_2_O_3_ (1 mL) and basified with a saturated solution of Na_2_CO_3_ to pH 10. The aqueous phase was extracted with CH_2_Cl_2_ (4 × 3 mL), and the combined organic phases were dried over Na_2_SO_4_ and evaporated to dryness under reduced pressure. The crude product was purified by column chromatography on silica gel using CH_2_Cl_2_/MeOH (100:0 to 95:5 *v*/*v* containing 1% NEt_3_) as eluent. **11** was obtained as a pale orange solid (28 mg, 0.070 mmol, 91% yield). ^1^H NMR (CDCl_3_, 400 MHz): δ 7.50 (s, 1H); 6.68 (s, 1H); 3.83 (s, 3H); 2.93 (d, 1H, *J* = 18.3); 2.82–2.80 (m, 1H); 2.56 (dd, 1H, *J* = 5.7, 18.1); 2.48–2.45 (m, 1H); 2.39 (s, 3H); 2.33–2.30 (m, 1H); 2.06 (td, 1H, *J* = 3.2, 12.4); 1.87–1.83 (m, 1H); 1.77 (td, 1H, *J* = 4.8, 12.7); 1.65–1.62 (m, 2H); 1.42–1.25 (m, 5H); 1.08 (qd, 1H, *J* = 3.7, 12.5). ^13^C NMR (CDCl_3_, 100 MHz): δ 157.0, 142.2, 138.5, 132.2, 108.2, 83.1, 58.0, 56.6, 47.3, 45.1, 42.8, 41.8, 37.5, 36.7, 26.8, 26.5, 23.2, 22.3. HRMS (ESI): *m*/*z* calculated for C_18_H_25_NOI^+^ [M + H^+^] = 398.0975. Found = 398.0973. The analytical data were in accordance with the literature [17].

**DX 12, (9α,13α,14α)-17-methyl-3-hydroxymorphinan, C_17_H_23_NO** (***O*****-Demethylation of DXM 3).** To a solution of aqueous HBr (48%, 8 mL), dextromethorphan **3** hydrobromide monohydrate (1.61 g, 4.35 mmol) was stirred in, and the resulting solution was heated to reflux overnight. The reaction mixture was cooled in an ice bath and basified to pH 8 by 2N NaOH aqueous solution, then extracted with CHCl_3_ (5 × 20 mL). The combined organic phases were dried over Na_2_SO_4_ and evaporated under a vacuum to produce DX **12** as a white solid (1.02 g, 3.96 mmol, 91% yield). An analytical sample was purified by column chromatography on silica gel using CH_2_Cl_2_/MeOH (98:2 to 97:3 *v*/*v* containing 1% NEt_3_) as eluent. ^1^H NMR (CDCl_3_, 300 MHz): δ 6.95 (d, 1H, *J* = 8.2); 6.70 (d, 1H, *J* = 2.6); 6.61 (dd, 1H, *J* = 2.6, 8.2); 2.99 (d, 1H, *J* = 18.2); 2.91–2.86 (m, 1H); 2.66 (dd, 1H, *J* = 5.8, 18.2); 2.56–2.47 (m, 1H); 2.41 (s, 3H); 2.87–2.16 (m, 2H); 1.94–1.86 (m, 1H); 1.77 (td, 1H, *J* = 4.2, 12.7); 1. 65–1.56 (m, 1H); 1.46–1.25 (m, 7H); 1.23–1.06 (m, 1H). The analytical data were in accordance with the literature [17].

**2-I-DX 13, (9α,13α,14α)-2-iodo-17-methyl-3-hydroxymorphinan, C_17_H_22_INO.** To a light-protected solution of **12** (21 mg, 0.082 mmol) in MeCN (1.5 mL) at 0 ${}^{\circ }$C, *N*-iodosuccinimide (20 mg, 0.089 mmol) was added, followed by *p*-toluenesulfonic acid monohydrate (27 mg, 0.14 mmol). The mixture was allowed to warm up to r. t. and was stirred overnight. The reaction mixture was treated with water (1.5 mL) and 1 M Na_2_S_2_O_3_ (1.5 mL), and basified with a solution of saturated Na_2_CO_3_ to pH 10. The aqueous phase was extracted with CH_2_Cl_2_ (4 × 1 mL), and the combined organic phases were dried over Na_2_SO_4_ and evaporated to dryness under reduced pressure. The crude product was purified by column chromatography on silica gel using CH_2_Cl_2_/MeOH (100:0 to 95:5 *v*/*v* containing 1% NEt_3_) as eluent to afford **13** (27 mg, 87%). ^1^H NMR (CDCl_3_, 400 MHz): δ 7.42 (s, 1H); 6.78 (s, 1H); 5.29 (ls, 1H); 2.95–2.91 (m, 2H); 2.66 (dd, 1H, *J* = 5.9, 18.6); 2.59–2.55 (m, 1H); 2.44 (s, 3H); 2.22–2.15 (m, 2H); 1.91–1.88 (m, 1H); 1.77 (td, 1H, *J* = 4.7, 12.9); 1.62–1.59 (m, 1H); 1.47–1.38 (m, 2H); 1.33–1.18 (m, 4H); 1.07 (qd, 1H, *J* = 3.7, 12.4). The analytical data were in accordance with the literature [17].

**(9α,13α,14α)-2′,2′,2′-trichloroethyl-17-carboxylate-3-methoxymorphinan 14, C_20_H_24_ NO_3_Cl_3_ (*****N*****-demethylation of DXM 3,*****step 1*****).** To a solution of DXM **3** free base (1.3 g, 4.8 mmol) in toluene (3 mL), 2′,2′,2′-trichloroethylchloroformate (800 μL, 5.8 mmol) was added, and the reaction mixture was heated under reflux for 2 h. The mixture was cooled down to r. t., then washed with 5% HCl (3 mL), then with water (3 mL). The organic layer was separated, dried over anhydrous Na_2_SO_4_, then evaporated under reduced pressure. The crude product was purified by column chromatography on silica gel using EtOAc/cyclohexane (10:90 *v*/*v*) as eluent to produce **14** (3.1 g, 92%) as a white solid. ^1^H NMR (CDCl_3_, 400 MHz): Amide rotamers [7.03 and 7.00 (d, 1H, *J* = 8.4)]; 6.83 (d, 1H, *J* = 2.4); 6.73 (dd, 1H, *J* = 2.4, 8.4); 4.85 (t, 1H, *J* = 6.5); 4.75 (q, 1H, *J* = 12.0); 4. 39 (t, 1H, *J* = 4.4); 3.92 (td, 1H, *J* = 4.5, 12.8); 3.79 (s, 3H); 3.13 (dd, 1H, *J* = 6.0, 18.1); 2.79–2.26 (m, 2H); 2.38 (m, 1H); 1.74–1.26 (m, 9H); 1.09 (m, 1H). ^13^C (CDCl_3_, 100 MHz): δ 158.6, 153.7, 140.2, 129.1, 128.1, 111.4, 96.0, 95.1, 55.3, 50.7, 44.0, 41.7, 38.9, 37.6, 36.5, 31.4, 26.6, 26.4, 22.1. The analytical data were in accordance with the literature [18,20].

***Nor*****-DXM 15, (9α,13α,14α)-3-hydroxymorphinan, C_17_H_23_NO (*****N*****-demethylation of DXM 3,*****step 2*****).** To a solution of (9α,13α,14α)-2′,2′,2′-trichloroethyl-17-carboxylate-3-methoxy morphinan **14** (3 g, 6.93 mmol) in aqueous AcOH (90%, 30 mL), zinc powder (1.36 g, 20.8 mmol) was added in several portions over 30 min. After 1 h of additional stirring, the zinc powder was filtered off using celite^®^, and the solvent evaporated under reduced pressure. Toluene (6 mL) was added to solubilize the obtained dense oil, the mixture was brought to reflux, then allowed to cool down to 4 ${}^{\circ }$C. The resulting white precipitate of (9α,13α,14α)-3-methoxymorphinan tetraacetozincate was filtered off and washed four times with Et_2_O (5 mL). The precipitate was then dissolved in CHCl_3_ (6 mL) and basified to pH 9.5 with 1 M NaOH aqueous solution. The obtained white suspension was shaken with CHCl_3_ (30 mL), filtered using fritted glass, and the organic layer was separated. The aqueous layer was further extracted with CHCl_3_ (4 × 10 mL), the organic layers were combined, dried over anhydrous Na_2_SO_4_, and the solvent was evaporated under reduced pressure to produce *nor*-DXM **15** (0.832 g, 3.23 mmol, 47% yield) as a clear oil that solidified upon standing. ^1^H NMR (CDCl_3_, 400 MHz): δ 9.55 (ls, 1H); 6.98 (d, 1H, *J* = 8.4); 6.69 (d, 1H, *J* = 2.6); 6.35 (dd, 1H, *J* = 2.6, 8.4); 3.67 (s, 3H); 3.63–3.58 (m, 1H); 3.19–3.03 (m, 3H); 2.63 (t, *J* = 12.0, 1H); 2.24 (d, 1H, *J* = 13.6); 2.08 (d, 1H, *J* = 12.0); 1.92 (td, 1H, *J* = 43.6, 13.6); 1.53 (d, 1H, *J* = 12.4); 1.46–1.25 (m, 5H); 1.21–1.12 (m, 1H); 0.96 (qd, 1H, *J* = 2.4, 12.4). The analytical data were in accordance with the literature [18,20].

**General procedure for the reductive animation of*****nor*****-DXM 15 (cpds. 16a–m).** To a stirred solution of *nor*-DXM **15** (30 mg, 0.117 mmol) in anhydrous DMF (300 μL), the corresponding aldehyde (0.129 mmol) was added at r. t. under an argon atmosphere. After 10 min, STABH (97%, 51 mg, 234 μmol) was added in one portion. The resulting suspension was stirred until completion (TLC monitoring, Table 1), then, H_2_O (100 μL) was added and the reaction mixture was partitioned in a system composed of EtOAc (300 μL) and NaHCO_3_/Na_2_CO_3_ buffer (pH 9.5, 300 μL). After separation, the organic phase was washed with carbonate buffer (3 × 300 μL), dried over Na_2_SO_4_, and concentrated under reduced pressure. The crude product was purified by column chromatography on silica gel using cyclohexane/EtOAc (95:5 to 90:10 *v*/*v* containing 1% NEt_3_) as eluent, yielding *N*-substituted (9α,13α,14α)-3-methoxymorphinans **16a**–**m** at 33–97%.

**Cpd. 16a, (9α,13α,14α)-17-*****n*****-propyl-3-methoxymorphinan, C_20_H_29_NO.**^1^H NMR (CDCl_3_, 600 MHz): δ 7.02 (d, 1H, *J* = 8.3); 6.80 (d, 1H, *J* = 2.4); 6.69 (dd, 1H, *J* = 2.4, 8.3); 3.79 (s, 3H); 3.78–3.95 (m, 2H); 2.61 (dd, 1H, *J* = 4.9, 17.9); 2.55 (d, 1H, *J* = 9.5); 2.51–2.45 (m, 2H); 2.34 (d, 1H, *J* = 13.3); 2.06 (t, 1H, *J* = 11); 1.86 (d, 1H, *J* = 10); 1.77 (t, 1H, *J* = 10.7); 1.63 (d, 1H, *J* = 12.1); 1.55–1.50 (m, 3H); 1.42–1.25 (m, 5H); 1.13 (qd, 1H, *J* = 3.5, 12.6); 0.93–0.90 (t, 3H, *J* = 7.2). ^13^C NMR (CDCl_3_, 150 MHz): δ 158.3, 141.9, 130.0, 128.6, 111.2, 110.8, 57.2, 56.1, 55.3, 46.0, 45.0, 42.0, 38.0, 36.7, 27.0, 26.7, 24.1, 22.4, 20.9, 12.2. HRMS (ESI): *m*/*z* calculated for C_20_H_30_NO^+^ [M + H]^+^ = 300.2322. Found = 300.2323.

**Cpd. 16b, (9α,13α,14α)-17-*****n*****-butyl-3-methoxymorphinan, C_21_H_31_NO.**^1^H NMR (CDCl_3_, 300 MHz): δ 7.02 (d, 1H, *J* = 9.0); 6.80 (d, 1H, *J* = 3.0); 6.69 (dd, 1H, *J* = 3.0, 9.0); 3.78 (s, 3H); 2.96–2.90 (m, 2H); 2.64–2.45 (m, 4H); 2.35–2.32 (m, 1H); 2.08–2.00 (td, 1H, *J* =3.0, 9.0); 1.87–1.72 (m, 2H); 1.64–1.61 (m, 1H); 1.54–1.45 (m, 3H); 1.44–1.27 (m, 7H); 1.13–1.08 (m, 1H); 0.94–0.90 (t, 3H, *J* = 6.0). ^13^C NMR (CDCl_3_, 75 MHz): δ 158.2, 141.8, 129.8, 128.5, 111.1, 110.7, 55.8, 55.2, 54.8, 45.9, 45.0, 41.9, 37.9, 36.6, 29.8, 26.9, 26.6, 23.9, 22.3, 21.0, 14.2. HRMS (ESI): *m*/*z* calculated for C_21_H_32_NO^+^ [M + H]^+^ = 314.2478. Found = 314.2480.

**Cpd. 16c, (9α,13α,14α)-17-*****n*****-pentyl-3-methoxymorphinan, C_22_H_33_NO.**^1^H NMR (CDCl_3_, 400 MHz): δ 7.00 (d, 1H, *J* = 8.3); 6.80 (d, 1H, *J* = 2.6); 6.69 (dd, 1H, *J* = 2.6, 8.3); 3.78 (s, 3H); 2.96–2.89 (m, 2H); 2.61 (d, 1H, *J* = 5.5); 2.56–2.44 (m, 3H); 2.33 (d, 1H, *J* = 12.3); 2.04–2.01 (m, 1H); 1.84 (d, 1H, *J* = 12.4); 1.76 (m, 1H); 1.63 (m, 1H); 1.54–1.47 (m, 3H); 1.41–1.26 (m, 9H); 1.13–1.09 (qd, 1H *J* = 2.8, 11.8); 0.89 (t, 3H, *J* = 6.8). ^13^C NMR (CDCl_3_, 100 MHz): δ 158.3, 142.0, 130.0, 128.6, 111.2, 110.8, 56.0, 55.3, 55.2, 46.0, 45.2, 42.0, 38.0, 36.8, 30.1, 27.5, 27.0, 26.7, 24.0, 22.8, 22.4, 14.2. HRMS (ESI): *m*/*z* calculated for C_22_H_34_NO^+^ [M + H]^+^ = 328.2635. Found = 328.2635.

**Cpd. 16d, (9α,13α,14α)-17-cyclopropylmethyl-3-methoxymorphinan C_21_H_29_NO.**^1^H NMR (CDCl_3_, 400 MHz): δ 7.00 (d, 1H, *J* = 8.4); 6.80 (d, 1H, *J* = 2.5); 6.69 (dd, 1H, *J* = 2.5, 8.4); 3.80 (s, 3H); 3.17–3.12 (m, 1H); 2.88 (d, 1H, *J* = 18.2); 2.77–2.71 (m, 1H); 2.65–2.60 (m, 1H); 2.55–2.50 (m, 1H); 2.35 (d, 2H, *J* = 13.0); 2.03 (t, 1H, *J* = 13.2); 1.91 (d, 1H, *J* = 9.6); 1.82 (t, 1H, *J* = 11.5); 1.64 (d, 1H, *J* = 13.0); 1.55–1.49 (m, 1H); 1.44–1.27 (m, 5H); 1.15 (qd, 1H, *J* = 2.4, 12.4); 0.95–0.87 (m, 1H); 0.55–0.50 (m, 2H); 0.16–0.12 (m, 2H). ^13^C NMR (CDCl_3_, 100 MHz): δ 158.6, 142.1, 130.1, 128.8, 111.4, 111.1, 60.2, 56.3, 55.5, 46.2, 45.3, 42.2, 38.2, 36.9, 27.3, 26.9, 24.3, 22.6, 4.5, 4.01. HRMS (ESI): *m*/*z* calculated for C_21_H_30_NO^+^ [M + H]^+^ = 312.2322. Found = 312.2341.

**Cpd. 16e, (9α,13α,14α)-17-cyclopentylmethyl-3-methoxy morphinan C_23_H_33_NO.**^1^H NMR (CDCl_3_, 600 MHz): δ 7.00 (d, 1H, *J* = 8.3); 6.80 (d, 1H, *J* = 2.5); 6.69 (dd, 1H, *J* = 2.2, 8.3); 3.78 (s, 3H); 2.92 (d, 1H, *J* = 17.7); 2.66–2.37 (m, 1H); 2.33 (d, 1H, *J* = 13.3); 2.13–2.00 (m, 2H); 1.86–1.71 (m, 4H); 1.66–1.56 (m, 4H); 1.54–1.50 (m, 4H); 1.42–1.20 (m, 8H); 1.10 (qd, 1H, *J* = 3.9, 12.5). HRMS (ESI): *m*/*z* calculated for C_21_H_30_NO^+^ [M + H]^+^ = 340.2635. Found = 340.2636.

**Cpd. 16f, (9α,13α,14α)-17-cyclohexylmethyl-3-methoxy morphinan, C_24_H_35_NO.**^1^H NMR (CDCl_3_, 600 MHz): δ 7.00 (d, 1H, *J* = 8.3); 6.80 (d, 1H, *J* = 2.3); 6.68 (dd, 1H, *J* = 2.3, 8.3); 3.80 (s, 3H); 2.91 (d, 1H, *J* = 17.9); 2.79–2.75 (m, 1H); 2.58 (d, 1H, *J* = 17.0); 2.45–2.40 (m, 1H); 2.34–2.25 (m, 3H); 2.07–2.01 (m, 1H); 1.81 (d, 1H, *J* = 12.2); 1.75–1.62 (m, 6H); 1.50 (d, 1H, *J* = 11.3); 1.40–1.33 (m, 11H); 0.93–0.82 (m, 2H). ^13^C NMR (CDCl_3_, 150 MHz): δ 158.2, 142.2, 130.0, 128.7, 111.2, 110.7, 62.3, 56.7, 55.3, 46.3, 45.3, 42.3, 38.4, 36.8, 35.9, 32.2, 27.1, 27.0, 26.8, 26.4, 24.6, 22.4. HRMS (ESI): *m*/*z* calculated for C_24_H_36_NO^+^ [M + H]^+^ = 354.2791. Found = 354.2790.

**Cpd. 16g, (9α,13α,14α)-17-(2${}^{\prime }$-furanylmethyl)-3-methoxy morphinan, C_22_H_27_NO_2_.**^1^H NMR (CDCl_3_, 400 MHz): δ 7.39–7.38 (m, 1H); 7.04 (d, 1H, *J* = 8.4); 6.81(d, 1H, *J* = 2.6); 6.70 (dd, 1H, *J* = 2.6, 8.4); 6.31–6.30 (m, 1H); 6.24–6.20 (m, 1H); 3.79 (s, 3H); 3.70 (q, 2H, *J* = 13.8); 2.98 (d, 1H, *J* = 18.3); 2.85–2.81 (m, 1H); 2.63 (dd, 1H, *J* = 5.3, 18.2); 2.55–2.50 (m, 1H); 2.33 (d, 1H, *J* = 12.8); 2.17–2.08 (m, 1H); 1.89 (d, 1H, *J* = 12.4); 1.83–1.73 (m, 1H); 1.63–1.60 (m,1H); 1.52–1.38 (m, 1H); 1.35–1.29 (m, 5H); 1.11 (qd, 1H, *J* = 3.6, 12.4). ^13^C NMR (CDCl_3_, 100 MHz): δ 158.3, 142.3, 140.0, 129.8, 128.6, 111.2, 110.8, 110.2, 108.5, 55.9, 55.3, 51.9, 45.8, 44.9, 41.8, 37.9, 36.7, 27.0, 26.9, 26.7, 24.1, 22.4. HRMS (ESI): *m*/*z* calculated for C_22_H_28_NO_2_^+^ [M + H]^+^ = 338.2115. Found = 338.2116.

**Cpd. 16h, (9α,13α,14α)-17-(2${}^{\prime }$-thiophenylmethyl)-3-methoxymorphinan, C_22_H_27_NOS.**^1^H NMR (CDCl_3_, 400 MHz): δ 7.22 (d, 1H, *J* = 3.6); 7.04 (d, 1H, *J* = 8.4); 6.92 (d, 1H, *J* = 2.4); 6.81 (d, 1H, *J* = 2.4); 6.71 (dd, 1H, *J* = 2.4, 8.4); 3.94–3.78 (m, 2H); 3.79 (s, 3H); 3.01–2.91 (m, 2H); 2.64 (dd, 1H, *J* = 4.6, 17.8); 2.55 (d, 1H, *J* = 9.2); 2.34 (d, 1H, *J* = 12.8); 2.13 (t, 1H, *J* = 11.7); 1.87 (d, 1H, *J* = 11.6); 1.79–1.68 (m, 1H); 1.62 (d, 1H, *J* = 9.2); 1.52 (d, 1H, *J* = 9.2); 1.41–1.24 (m, 6H); 1.09 (qd, 1H *J* = 3.4, 12.5). ^13^C NMR (CDCl_3_, 100 MHz): δ 158.4, 142.1, 130.1, 128.6, 128.0, 126.5, 125.1, 124.8, 111.3, 110.8, 56.0, 55.3, 54.1, 45.6, 45.1, 42.1, 38.0, 36.7, 26.9, 26.8, 24.7, 22.4. HRMS (ESI): *m*/*z* calculated for C_22_H_28_NOS^+^ [M + H]^+^ = 354.1885. Found = 354.1885.

**Cpd. 16i, (9α,13α,14α)-17-(2${}^{\prime }$-pyrrolylmethyl)-3-methoxy morphinan, C_22_H_28_N_2_O.**^1^H NMR (CDCl_3_, 400 MHz): δ 8.75 (ls, 1H); 7.04 (d, 1H, *J* = 8.4); 6.81 (d, 1H, *J* = 2.4); 6.75 (q, 1H, *J* = 1.4); 6.70 (dd, 1H, *J* = 2.6, 8.4); 6.12 (q, 1H, *J* = 2.8); 6.03–6.00 (m, 1H); 3.79 (s, 3H); 3.70 (q, 2H, *J* = 13.8), 2.97 (d, 1H, *J* = 18.1); 2.83–2.80 (m, 1H), 2.63 (dd, 1H, *J* = 5.8, 18.2); 2.46 (dd, 1H, *J* = 3.2, 12.0); 2.38–2.33 (m, 1H); 2.13 (td, 1H, *J* = 3.0, 12.4); 1.85–1.79 (m, 1H); 1.72–1.62 (m, 2H); 1.55–1.49 (m, 1H); 1.38–1.30 (m, 5H); 1.15–1.09 (m, 1H). ^13^C NMR (CDCl_3_, 100 MHz): δ 158.3, 141.9, 129.9, 129.4, 128.6, 117.3, 111.3, 110.8, 108.0, 106.9, 55.8, 55.3, 52.0, 45.6, 45.3, 42.1, 38.0, 36.8, 26.9, 26.7, 24.5, 22.4. HRMS (ESI): *m*/*z* calculated for C_22_H_29_N_2_O^+^ [M + H]^+^ = 337.2274. Found = 337.2275.

**Cpd. 16j, (9α,13α,14α)-17-(*****N*****-methyl-2${}^{\prime }$-pyrrolylmethyl)-3-methoxymorphinan, C_23_H_30_N_2_O.**^1^H NMR (CDCl_3_, 600 MHz): δ 7.06 (d, 1H, *J* = 8.3); 6.81 (d, 1H, *J* = 1.9); 6.72 (dd, 1H *J* = 1.9, 8.3); 6.60 (s, 1H); 6.02 (s, 1H); 5.97 (s, 1H); 3.78 (s, 3H); 3.68–3.54 (m, 5H); 3.99 (d, 1H, *J* = 18.0); 2.79–2.76 (m, 1H); 2.62–2.57 (m, 1H); 2.46–2.43 (m, 1H); 2.36–2.33 (m, 1H); 2.09–2.04 (m, 1H); 1.76–1.73 (m, 1H); 1.65–1.62 (m, 2H); 1.52–1.50 (m, 1H); 1.39–1.29 (m, 5H); 1.18–1.07 (m, 1H). ^13^C NMR (CDCl_3_, 150 MHz): δ 158.3, 142.1, 130.3, 130.0, 128.6, 122.5, 111.3, 110.7, 109.0, 106.1, 55.4, 55.3, 53.5, 51.2, 45.5, 45.1, 42.3, 38.0, 36.8, 34.0, 26.7, 24.2, 22.4. HRMS (ESI): *m*/*z* calculated for C_23_H_31_N_2_O^+^ [M + H]^+^ = 351.2431. Found = 351.2431.

**Cpd. 16k, (9α,13α,14α)-17-(4″-methyl-5${}^{\prime }$-imidazolylmethyl)-3-methoxymorphinan, C_22_H_29_N_3_O.**^1^H NMR (CDCl_3_, 400 MHz): δ 7.44 (s, 1H); 7.00 (d, 1H, *J* = 8.4); 6.74 (d, 1H, *J* = 2.4); 6.45 (dd, 1H, *J* = 2.4, 8.4); 3.72 (s, 3H), 3.63 (q, 2H, *J* = 13.6); 2.92 (d, 1H, *J* = 18.4); 2.82–2.78 (m, 1H); 2.63 (dd, 1H, *J* = 5.2, 18.0); 2.53–2.45 (m, 1H); 2.30–2.26 (m, 1H); 2.15 (s, 3H); 1.81–1.78 (m, 1H); 1.71–1.66 (m, 1H); 1.58–1.56 (m, 1H); 1.47–1.44 (m, 1H); 1.36 (s, 3H); 1.30–1.19 (m, 4H); 1.17–0.98 (m, 1H). HRMS (ESI): *m*/*z* calculated for C_22_H_30_N_3_O^+^ [M + H]^+^ = 352.2383. Found = 352.2378.

**Cpd. 16l, (9α,13α,14α)-17-(2${}^{\prime }$-indolylmethyl)-3-methoxymorphinan, C_26_H_30_N_2_O.**^1^H NMR (CDCl_3_, 400 MHz): δ 8.79 (ls, 1H); 7.54 (d, 1H, *J* = 8.0); 7.36 (d, 1H, *J* = 8.0); 7.15 (t, 1H, *J* = 7.2); 7.07 (t, 1H, *J* = 6.8); 6.81 (d, 1H, *J* = 2.0); 6.72 (dd, 1H, *J* = 2.4, 8.4); 6.33 (s, 1H); 3.88 (q, 2H, *J* = 13.6); 3.81 (s, 3H); 3.01 (d, 1H, *J* = 18.4); 2.87–2.43 (m, 1H), 2.67 (dd, 1H, *J* = 5.6, 18.0); 2.51 (dd, 1H, *J* = 3.2, 12.0); 2.33 (d, 1H, *J* = 12.8); 2.21 (td, 1H, *J* = 3.2, 12.0); 1.91–1.87 (m, 1H); 1.74 (td, 1H, *J* = 3.2, 12.4); 1.65–1.62 (m, 1H); 1.54–1.51 (m, 1H); 1.40–1.32 (m, 5H); 1.26 (t, 1H, *J* = 7.2); 1.15–1.05 (m, 1H). ^13^C NMR (CDCl_3_, 100 MHz): δ 158.3, 136.1, 128.6, 128.5, 121.5, 120.1, 119.6, 111.2, 110.8, 56.0, 55.2, 52.3, 45.7, 41.8, 37.8, 36.6, 26.7, 26.5, 24.6, 22.2. HRMS (ESI): *m*/*z* calculated for C_26_H_31_N_2_O^+^ [M + H]^+^ = 387.2436. Found = 387.2425.

**Cpd. 16m, (9α,13α,14α)-17-(5${}^{\prime }$-(2${}^{\prime }$,2″-bithiophenyl)methyl)-3-methoxymorphinan, C_26_H_29_NOS_2_.**^1^H NMR (CDCl_3_, 400 MHz): δ 7.18 (d, 1H, *J* = 5.2); 7.14 (d, 1H, *J* = 3.2); 7.06 (d, 1H, *J* = 8.4); 7.01–6.98 (m, 2H); 6.82 (d, 1H, *J* = 2.0); 6.72 (dd, 1H, *J* = 2.4, 8.4); 3.92–3.81 (m, 2H); 3.82 (s, 3H); 2.99–2.95 (m, 2H); 2.71–2.66 (m, 2H); 2.35 (d, 1H, *J* = 12.8); 2.07–2.03 (m, 1H); 1.92–1.89 (m, 1H); 1.82–1.78 (m, 1H); 1.64–1.62 (m, 1H); 1.54 (d, 1H, *J* = 11.6); 1.41–1.24 (m, 6H); 1.10 (qd, 1H, *J* = 4.0, 12.4). HRMS (ESI): *m*/*z* calculated for C_26_H_30_NOS_2_^+^ [M + H]^+^ = 436.1769. Found = 436.1757.

**Cpd. 17, (9α,13α,14α)-17-cyclopropylcarbonyl-3-methoxymorphinan, C_21_H_27_NO_2_.** To solution of *nor*-DXM **15** (36 mg, 0.140 mmol) in CH_2_Cl_2_ (500 μL) at 0${}^{\circ }$C was added cyclopropanecarbonyl chloride (16 mg, 0.153 mmol) then NEt_3_ (20 μL). The reaction mixture was allowed to warm to r. t. and further stirred for 1.5 h. The mixture was treated with NaHCO_3_/Na_2_CO_3_ buffer (pH 9.5, 500 μL), the organic layer was separated, dried over MgSO_4_, filtered and evaporated under reduced pressure. The crude product was purified by column chromatography on silica gel using EtOAc/cyclohexane (0:100 to 20:80 *v*/*v*) as eluent to afford **17** (34 mg, 75%).^1^H NMR (CDCl_3_, 400 MHz): δ 7.02 (d, 1H, *J* = 8.4); 6.84 (d, 1H, *J* = 2.6); 6.72 (dd, 1H, *J* = 2.6, 8.4); 4.76–4.65 (m, 1H); 4.09–4.03 (m, 1H,); 3.79 (s, 3H); 3.13 (dd, 1H, *J* = 6.0, 18.0); 2.66 (d, 1H, *J* = 17.6); 2.40–2.36 (m, 1H); 1.74–1.24 (m, 11H); 1.14–1.04 (m, 1H); 0.98–0.94 (m, 1H); 0.76–0.71 (m, 1H). ^13^C NMR (CDCl_3_, 100 MHz): δ 171.9, 158.6, 140.6, 129.2, 128.5, 111.5, 111.3, 55.4, 44.2, 42.1, 38.0, 36.6, 31.6, 26.7, 22.2, 11.6, 7.4, 7.2. HRMS (ESI): *m*/*z* calculated for C_21_H_27_NO_2_Na^+^ [M + Na]^+^ = 348.1934. Found = 348.1935.

**Cpd. 18, (9α,13α,14α)-17,17-dimethyl-3-methoxy morphinan iodide, C_19_H_28_NOI.** To a solution of DXM **3** free base (30 mg 0.11 mmol) in CH_2_Cl_2_ (300 μL), CH_3_I (300 μL, 4.82 mmol) was added, and the mixture was stirred for 2 h at r. t. The obtained white precipitate was filtered and washed with EtOAc (4 × 300 μL) to produce **18** as a white powder (26 mg, 0.091 mmol, 82% yield). ^1^H NMR (CDCl_3_, 400 MHz): δ 7.12 (d, 1H, *J* = 9.2); 6.80–6.77 (m, 2H); 4.10–4.07 (m, 1H); 3.58 (s, 3H); 3.69 (s, 3H); 3.61–3.55 (m, 1H); 3.52 (s, 3H); 3.38–3.32 (m, 1H); 2.87–2.85 (m, 1H); 2.43–2.36 (m, 2H); 2.14–2.12 (m, 1 H); 1.74–1.44 (m, 6H); 1.33–1.22 (m, 1H); 1.12 (qd, 1H, *J* = 3.6, 12.4). ^13^C NMR (CDCl_3_, 100 MHz): δ 159.7, 138.6, 129.5, 124.1, 112.6, 111.4, 69.6, 57.7, 55.5, 54.5, 51.2, 38.7, 36.8, 36.5, 35.5, 26.7, 26.4, 25.9, 21.8. HRMS (ESI): *m*/*z* calculated for C_19_H_28_NO^+^ [M^+^] = 286.2171. Found = 286.2166.

**Drug preparation and storage**. Tazopsine **1**, DXM **3**, and derivatives (cpds. **6**–**18**) were prepared in DMSO at 10 mM, aliquoted, then stored at −20 ${}^{\circ }$C. PQ and CQ biphosphates were prepared at 110 mM and 132 μM, respectively, in sterile water, aliquoted, then stored at −20 ${}^{\circ }$C. A stock aliquot was thawed and used for daily medium changes of the parasite cultures whenever necessary.

### 2.3. Parasite Maintenance and Inhibition Assays (By Order of Appearance in the Manuscript)

#### 2.3.1. *P. yoelii* Growth Inhibition Assays In Vitro

**Parasite culture.** Primary mouse hepatocytes were isolated as previously described [21] and seeded at 10^5^ cells per well in eight-well Lab-Tek plastic chamber slides (VWR, Fontenay-sous-Bois, France) previously coated with rat tail collagen I (BD Biosciences, Le Pont de Claix, France). Mouse hepatocytes were cultured at 37 ${}^{\circ }$C in 5% CO_2_ in complete William’s E medium (10% fetal calf serum, 1% l-glutamine, 1% sodium pyruvate, 1% insulin-transferrin-selenium, 1% non-essential amino acids, and 1% penicillin-streptomycin, all obtained from Invitrogen, Cergy-Pontoise, France). After 24 h, 10^5^*P. yoelii* (265 BY strain) sporozoites, isolated from infected *Anopheles stephensi*, were added per well. Compounds to be tested were solubilized in DMSO, further diluted in complete William’s E medium (final DMSO concentrations < 0.3%), and added to hepatocyte cultures at the time of sporozoite inoculation. Each cpd. concentration was tested in triplicate. Infected cultures were maintained for 1 h at r. t. to allow parasites to settle down onto the hepatocytes, and were then transferred to the incubator at 37 ${}^{\circ }$C in an atmosphere containing 5% CO_2_. After 3 h, cultures were washed and further incubated in the presence of each test cpd. Culture medium containing the appropriate cpd. concentration was renewed 24 h later, and cultures were fixed with cold methanol 48 h post-infection.

**IC_50_ measurement.** After fixation of hepatocyte cultures with cold methanol, parasites were stained with an anti-*P. falciparum* heat shock protein 70 mouse polyclonal serum (cross-reactive with *P. yoelii*) and revealed with FITC-conjugated goat anti-mouse IgG secondary antibody (Sigma-Aldrich). Parasites were counted under a fluorescence microscope with a 25× light objective. IC_50_ values, e.g., compound concentrations at which a 50% reduction in parasite number was observed compared to DMSO control, were calculated by linear regression using Excel 2016 software and derived from three independent experiments.

#### 2.3.2. *P. yoelii* Growth Inhibition Assays In Vivo

These experiments were performed as described in Bosson Vanga et al. [22]. Briefly, 6- to 8-week-old BALB/c female mice were used (Janvier CERJ, Le Genest-Saint-Isle, France) and housed at CEF (UMS28, La Pitié-Salptrière). All animal work was conducted in strict accordance with the European Parliament and Council Directive 2010/63/EU on the protection of animals used for scientific purposes. Protocols were approved by the Charles Darwin Ethics Committee for animal experimentation CEEA-005 (approval #01736.02). Five mice were used per treatment group. Drugs were administrated on days −1, 0, +1, and mice were infected on day 0 by intravenous injection of 10,000 *P. yoelii*-Luc sporozoites. In vivo imaging was performed 44 h post-infection to assess liver stage development with an IVIS Spectrum (Caliper Life Science, Hanover, MD, USA). Mice were injected intraperitoneally with d-luciferin (100 mg/kg), anesthetized with isoflurane, and imaged for bioluminescence 10 min post-luciferin injection. Data acquisition was conducted using Living Image software 3.0 (Caliper Life Sciences, Hanover, MD, USA). Data analysis and statistical analysis using a one-way ANOVA test for multiple comparisons were performed with GraphPad Prism 8 statistical software (GraphPad. Software, San Diego, CA, USA).

#### 2.3.3. *P. falciparum* and *P. berghei* Liver Stages Growth Inhibition Assays In Vitro

**Parasite culture.***Plasmodium* liver stages were cultured as described elsewhere (Baron et al. manuscript in preparation). Briefly, cryopreserved primary human hepatocytes were purchased from Lonza Bioscience and Biopredic International (Saint-Grégoire, France). Cells were thawed and seeded into 384-well plates (Greiner Bio-One, Germany) pre-coated with rat tail collagen I (BD Bioscience, Le Pont de Claix, France). Human hepatocytes were maintained at 37 ${}^{\circ }$C in 5% CO_2_ in William’s E medium (Gibco) supplemented with 10% fetal clone III serum (HyClone, Dutscher, Bernolsheim, France), 100 μg/mL penicillin and 100 μg/mL streptomycin (ThermoFisher Scientific, Courtaboeuf, France), 5 × 10^−3^ g/L human insulin (Sigma-Aldrich, Saint Quentin Fallavier, France), and 5 × 10^−5^ M hydrocortisone (Upjohn Laboratories SERB, France). The next day, cells were overlaid with matrigel (Corning) and the medium was then renewed every two days. Four days later, sporozoites were isolated by aseptic hand dissection of salivary glands of *P. berghei*-GFP [23] or *P. falciparum*-infected mosquitoes (*P. falciparum* NF54 strain, obtained from the Department of Medical Microbiology, University Medical Centre, St Radboud, Nijmegen, The Netherlands). Matrigel was then removed from the hepatocyte culture, and 5000 or 30,000 sporozoites of *P. berghei*-GFP or *P. falciparum*, respectively, were inoculated into cells before centrifugation at 560 xg for 10 min at r. t. and subsequent incubation at 37 ${}^{\circ }$C and 5% CO_2_. Drugs were tested in quadruplicate, starting from time of sporozoite addition. After 3 h, infected cultures were covered with matrigel prior to addition of fresh cell culture medium containing the appropriate drug dilutions. Media, containing drugs or not, were renewed on a daily basis until cell fixation, which occurred at 48 h and 6 days post-infection for *P. berghei* and *P. falciparum* sporozoites, respectively.

**Immunostaining of liver stages.** Infected cultures were fixed using 4% paraformaldehyde (PFA) for 15 min at r. t., and liver stage parasites were immune-labeled with polyclonal anti-PfHSP70 murine serum and revealed with Alexa-Fluor 488-conjugated goat anti-mouse immunoglobulin (Invitrogen). DAPI was used to visualize nuclei.

**Parasite enumeration and toxicity assessment using high-content imaging.** A CellInsight High-Content Screening Platform and Studio HCS software (Thermo Fisher Scientific, Waltham, MA, USA) were used to determine parasite number and size in fixed cultures. Reduction in parasite size was calculated based on the average object area, as described previously [24]. Compound cytotoxicity was determined by counting DAPI-positive host cell nuclei.

**IC_50_ measurement.** IC_50_ values were determined by non-linear regression with GraphPad Prism 8 software. The logarithm of concentration was expressed as a function of the parasites number normalized to the drug free controls. The tests on 384-well plates were conducted in quadruplicates.

**Statistical Analysis.** GraphPad Prism 8 statistical software (GraphPad. Software, San Diego, CA, USA) and Excel 2016 software (Microsoft Office) were used in this study for the data analysis. All graph values represent means and error bars represent standard deviations (s. d.).

#### 2.3.4. *P. falciparum* Asexual Blood Stages

**Parasite culture.** Chloroquine-sensitive (3D7) *P. falciparum* strain was obtained from the Malaria Research and Reference Reagent Resource Center (MR4). Parasites were maintained in human erythrocytes (O^+^, provided by Etablissement français du sang, EFS, Rungis, France), at 5% hematocrit, suspended in complete culture medium RPMI 1640 supplemented with 25 mM HEPES, 20 mM d-glucose, 25 mM sodium bicarbonate, 0.4 mM hypoxanthine, 5 mM l-glutamine, and 10% AB human serum. Parasite cultures were maintained at 37 ${}^{\circ }$C in a gaseous environment composed of 5% CO_2_, 10% O_2_, and 85% N_2_. The culture medium was changed daily. Parasitemia was controlled using light microscopy (Axioskop microscope, ZEISS, Oberkochen, Germany) under oil immersion, after fixing thin blood smears with methanol and staining with Diff-Quik^TM^ stain set (RAL Diagnostics, Martillac, France).

**IC_50_ measurement.** A 50% inhibitory concentrations (IC_50_) determination test was carried out using isotopic ^3^H-hypoxanthine incorporation assays, as previously described [25], with minor modifications. Briefly, *P. falciparum* cultures at ring stage were highly synchronized by two consecutive treatments with 5% sorbitol (Sigma-Aldrich) in PBS (*v*/*v*) at 40 h intervals and diluted down to 0.3–0.5% parasitemia and 2% hematocrit. Parasites were dispensed into 96-well plates containing 14 serially diluted drug concentrations ranging from 0 to 240 μM, and incubated as described above in the presence of 5% ^3^H-hypoxanthine (Perkin Elmer, Waltham, Massachusetts, USA) for 42 h. Next, ^3^H-hypoxanthine uptake was evaluated by scintillation counting (Top Count NXT, Perkin Elmer, Waltham, Massachusetts, USA) and results were expressed as the inhibitory concentrations (IC_50_) defined as drug concentrations at which 50% of ^3^H-hypoxanthine incorporation was inhibited compared with drug-free controls. IC_50_ values were established by non-linear regression with ICEstimator software (http://www.antimalarial-icestimator.net/ 28 December 2021) [26,27]. The tests on 96-well plates were conducted in triplicate.

#### 2.3.5. *P. falciparum* Sexual Blood Stages

**Parasite culture and gametocyte production.** The *P. falciparum* transgenic line NF54-cg6-Pfs16-CBG99 has been described elsewhere [28,29]. Parasite cultures were grown in human erythrocytes at 5% hematocrit and RPMI 1640 media supplemented with hypoxanthine and 10% heat-inactivated human serum. Synchronization of asexual stages was achieved by magnetic isolation of schizonts from the culture, followed by depletion of schizonts several hours later, using a MACS depletion column (Miltenyi Biotec) in conjunction with a magnetic separator. To obtain synchronous gametocytes, cultures at 10–15% ring stages were treated with 50 mM N-acetylglucosamine (NAG) for 5 days to eliminate asexual parasites.

**IC_50_ measurement.** To calculate the IC_50_ for DXM **3** and *N*-2${}^{\prime }$-pyrrolylmethyl-*nor*-DXM **16i** on early and mature gametocytes, 2 × 10^5^ MACS-purified early GIE (day 2 post-NAG treatment) and mature GIE (day 7 post-NAG treatment) from the NF54-cg6-Pfs16-CBG99 line were incubated with serial dilutions of inhibitors, or 2% DMSO, for 72 h. After 72h, GIE were washed and cell viability was evaluated by adding a non-lysing formulation of 0.5 mM d-luciferin substrate [25], and by measuring luciferase activity for 1 s on an Infinite 200 PRO plate reader (Tecan^®^). The tests on 96-well plates were performed in triplicate.

## 3. Results

### 3.1. Extended SAR in the Tazopsine Series

**Figure 2:**
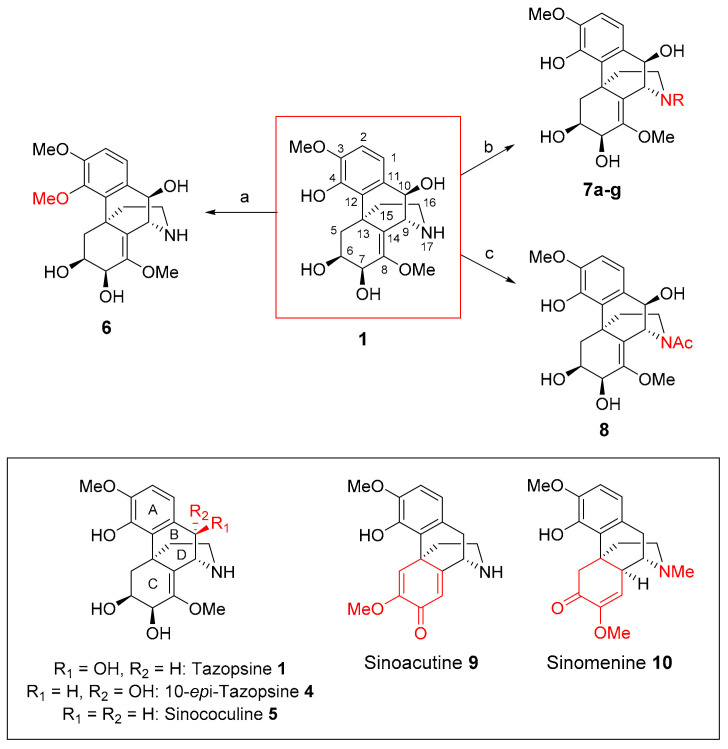
Semisynthetic access to tazopsine derivatives **6**–**8** and structure of natural *ent*-morphinan alkaloids for SAR generation (morphine numbering). (a) CH_2_N_2_, MeOH, 0 ${}^{\circ }$C, 12 h (39%); (b) aldehyde (1.1 eq), MeOH, r. t., 10 min then NaBH_3_CN (1 eq), r. t. (45–82%); (c) Ac_2_O (1 eq), MeOH, r. t., 1 h (45%). The box indicates the natural alkaloids.

We present here pharmacomodulation efforts towards novel derivatives of tazopsine **1**. Altogether, these SARs drove the validation of DXM **3** as a general and simplified mimic of all natural *ent*-morphinan alkaloids, and subsequently guided its chemical diversification into optimized antiplasmodial derivatives. Tazopsine **1** was treated with excess diazomethane to produce the 4-methyl phenol ether **6** with a 39% yield. The native alkaloid was, in parallel, submitted to reductive amination with various aldehydes in the presence of sodium cyanoborohydride, to deliver tertiary amines **7a**–**g** with 45–82% yields. To assess the influence of a basic nitrogen on the antiplasmodial activity, *N*-acetyl-tazopsine **8** was produced in a 45% yield by treating tazopsine **1** with acetic anhydride (Figure 2).

Primary mouse hepatocytes (PMH) infected by the murine parasite *P. yoelii* were used to assess the bioactivity of the generated tazopsine derivatives (Table 2), allowing a direct comparison with previously generated SARs in the series [14,15]. ***Benzylic 10-substitution (ring B) SAR:*** The comparison of tazopsine **1**, 10-*epi*-tazopsine **4**, and sinococuline **5** to assess the effect of benzylic 10-substitution, showed that the 10-(*R*)-hydroxy pattern of tazopsine **1** was optimal, its 10-epimer **4** being 5-fold less active. However, an unsubstituted benzylic position proved to be only slightly less beneficial than when 10-(*R*) was hydroxylated, as showed by the comparable bioactivities of sinococuline **5** and tazopsine **1**. ***Aromatic 4-O-substitution (ring A) SAR:*** The alkylation of the tazopsine **1** free phenol abolished the antiplasmodial activity, with 4-*O*-methyl-tazospine **6** showing non-significant inhibitory effects against the parasite even at 100 μM. ***17-N-substitution (ring D) SAR:*** The tertiary amine derivatives of tazopsine **1** showed reduced activity with the increase in substituent size, *N*-methyl-tazopsine **7a** already being 2-fold less active than the parent alkaloid, whereas the *N*-3${}^{\prime }$,4${}^{\prime }$-methylenedioxybenzyl congener exhibited abolished activity. However, this trend was mitigated by the beneficial *N*-4${}^{\prime }$-halo-benzyl substituents in analogues **7f** and **7g**, these exhibiting similar levels of inhibition to *N*-methyl-tazopsine **7a**. This suggests that specific substituents can favorably impact the antiplasmodial activity of *N*-modified *ent*-morphinans despite relative bulkiness, as previously observed in *N*-cyclopentyltazopsine **2** (IC_50_ = 3.5 ± 0.1 μM) [14]. On the other hand, *N*-acetyl-tazopsine **8** was completely devoid of activity, suggesting that the presence of a nitrogen atom, either basic (i.e., protonated at physiological pH values) or capable of engaging donating hydrogen bonds, was important for the antiplasmodial properties. ***Cyclohexenediol*****/*****cyclohexadienone*****/*****cyclohexenone (ring C) SAR:*** The comparison of tazopsine **1** with sinoacutine **9** and sinomenine **10** revealed that the southern portion of these alkaloids exerted a profound influence on their bioactivity. Indeed, only the 6,7-dihydroxy-8,14-methylenol moiety of tazopsine **1** correlated with strong antiplasmodial effects, while the distinctive methoxy-enone/dienone systems present in **9** and **10** led to abolished activity (Figure 2). In conclusion, regarding these antiplasmodial SARs in the *ent*-morphinan series, it appeared that the benzylic substitution at C-10 in ring B had to be either (*R*)-hydroxyl (as in tazopsine **1**) or non-existent (as in sinococuline **5** and DXM **3**). The ring A in tazopsine **1** seems to be a sensitive component to modify, considering the loss of activity exhibited by 4-*O*-methyltazopsine **6**. However, this punctual variation precludes a definitive conclusion. *N*-alkylation in the tazopsine series consistently appeared as a relevant pharmacomodulation at ring D, with frequent conservation of antiplasmodial activity. Lastly, the SAR regarding the southern ring C of *ent*-morphinans remains inconclusive, except for the restricted benefit of that present in tazopsine **1**, a fact corroborated by the previous description of bioactivity loss in the tazopsine-6,7-acetonide [14].

### 3.2. DXM Repurposing against Malaria

DXM **3** is a well-known antitussive drug and pain reliever, also used as a dissociative anesthetic and hallucinogen in recreational use [30,31]. Its pharmacology in the central nervous system is well established. Despite being considered a synthetic opiate, DXM **3** does not act at the level of the opioid receptors, binding instead with high affinity to sigma receptors as an agonist, and to a lesser extent to the phencyclidine channel of *N*-methyl-d-aspartate (NMDA) receptors as an antagonist [31]. The relationship between the neuropharmacology and the antitussive effects of DXM **3** is poorly understood [30]. The similarity of DXM **3** with tazopsine **1** regarding their *ent*-morphinan backbone prompted us to evaluate its antiplasmodial properties both in vitro and in vivo, having in mind its possible direct repurposing against malaria. In a preliminary screening against primary human hepatocytes (PHH) infected by *P. falciparum* in vitro, DXM **3** exhibited an activity that was only 2-fold less than that of tazopsine **1** (Table 3). It is noteworthy that the IC_50_ value of tazopsine **1** was double the previously described value of ca. 4 μM in this same biological model [14]. This observation can be explained by the shift in the PHH used for *P. falciparum* culture from clinical samples in the previous study to standardized, commercially available cryopreserved PHH in the present work. The whole assay was validated by the expected submicromolar activity of the reference drug primaquine (PQ) [14].

**Figure 3:**
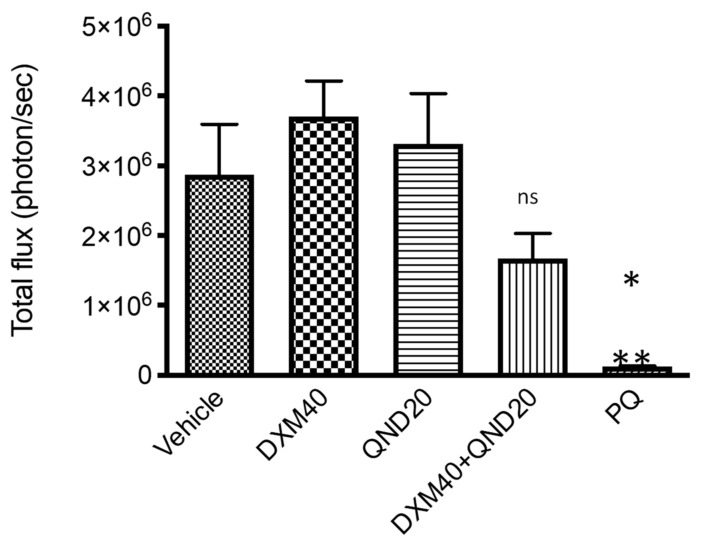
In vivo prophylactic activity of DXM **3** +/− QND compared to PQ in a mouse model of *P. yoelii*-Luc infection. Each group of mice consisted of five individuals. Results are shown as mean +/− SEM: Vehicle vs. DXM40 + QND20, *p* = 0.5171 (ns, not significative); Vehicle vs. PQ, *p* = 0.0124 (*); DXM40 vs. PQ and QND20 vs. PQ, *p* = 0.003 (**). ANOVA one-way test for multiple comparisons. Numeric values indicate doses in mg/kg.

Following the exciting discovery of the antiplasmodial activity of DXM **3** against *P. falciparum* liver stages in vitro, we tested its prophylactic potential in a *P. yoelii*-infected mouse model of malaria. We used the transgenic parasite line *P. yoelii*-Luc infecting BALB/c mice, to follow parasitemia in situ based on the spontaneously emitted bioluminescence after injection of d-luciferin [32]. Following a preliminary study, we found that DXM **3** induced convulsive episodes and death in mice at doses higher than 60 mg/kg administered daily (data not shown). Therefore, we decided to use a subtoxic regimen of 40 mg/kg DXM **3** administered daily, starting 24 h before infection and further maintained for a 48 h period, corresponding to the duration of *P. yoelii* liver phase. In addition, we chose to associate DXM **3** with quinidine (QND), a known inhibitor of CYP2D6-mediated *O*-demethylation of DXM **3** into its main hepatic metabolite dextrorphan **12** (DX, *syn.* 3-hydroxy-17-methylmorphinan) [31], in order to increase the plasmatic half-life of DXM **3** [33,34] and possibly increase its antimalarial effect in vivo. DXM **3** at 40 mg/kg and QND at 20 mg/kg were deprived of prophylactic activity in this in vivo model, with parasitemia levels not significatively different between these groups and the vehicle (Figure 3). Although the combination of DXM **3** and QND at a daily regimen of 40 mg/kg and 20 mg/kg, respectively, exerted significative prophylactic inhibition of *P. yoelii*-Luc growth, we found that this synergistic association was far from eliciting complete parasite clearance (vehicle vs. DXM40 + QND20: *p* = 0.5171), as achieved by the reference drug primaquine biphosphate at a daily regimen of 5 mg/kg (vehicle vs. PQ: *p* = 0.0124) (Figure 3).

**Figure 4:**
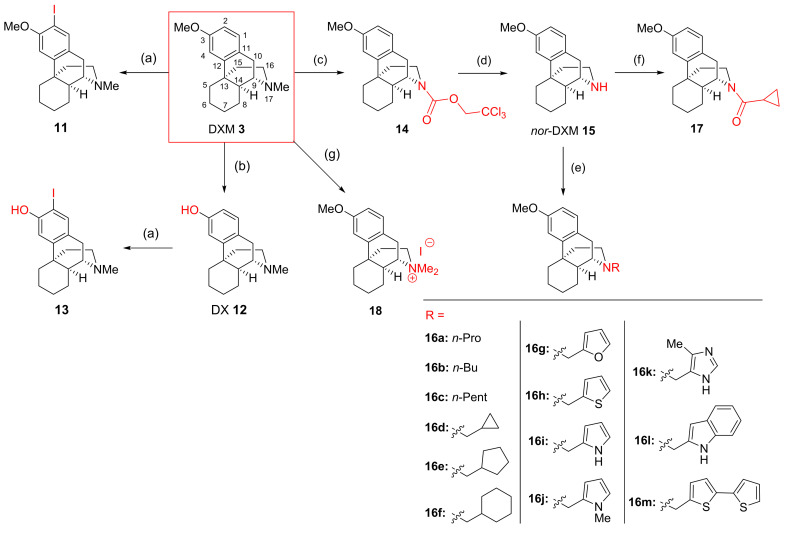
Semisynthetic access to DXM derivatives **11**–**18** for SAR generation (morphine numbering). (a) NIS (1.15 eq), *p*-TsOH (1.8 eq) (91% **11**, 87% **13**); (b) HBr 48%, reflux overnight (91%); (c) 2′,2′,2′-trichloroethylchloroformate (1.1 eq), toluene, reflux, 2 h (92%); (d) Zn powder (3 eq), AcOH, r. t., 1 h (47%); (e) aldehyde (1.1 eq), DMF, r. t., 10 min then STABH (2 eq), r. t. (33–97%); (f) cyclopropanecarbonyl chloride (1.1 eq), CH_2_Cl_2_, Et_3_N, 0 ${}^{\circ }$C to r. t, 1.5 h (75%); (g) MeI, rt, 2 h (82%).

**Figure 5:**
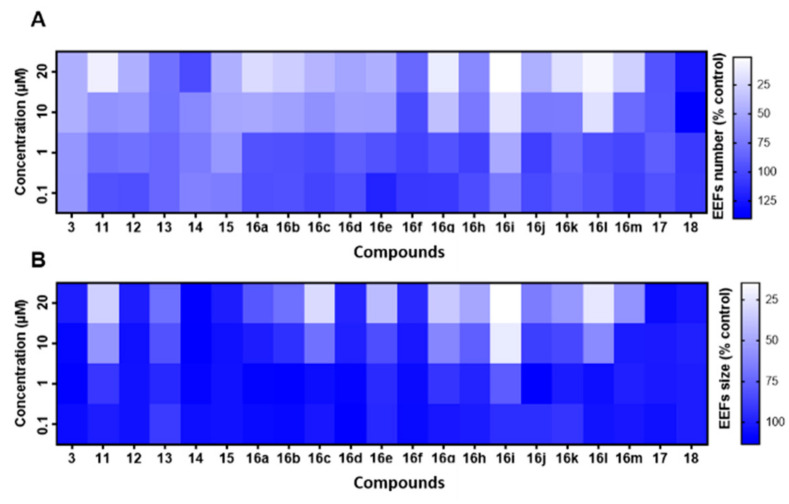
Normalized dose–response heatmap in PHH-*Pb*-GFP regarding EEF number (**A**) and EEF size (**B**) at 0.1, 1, 10, or 20 μM of the indicated compounds. These results were obtained from four technical replicates.

**Figure 6:**
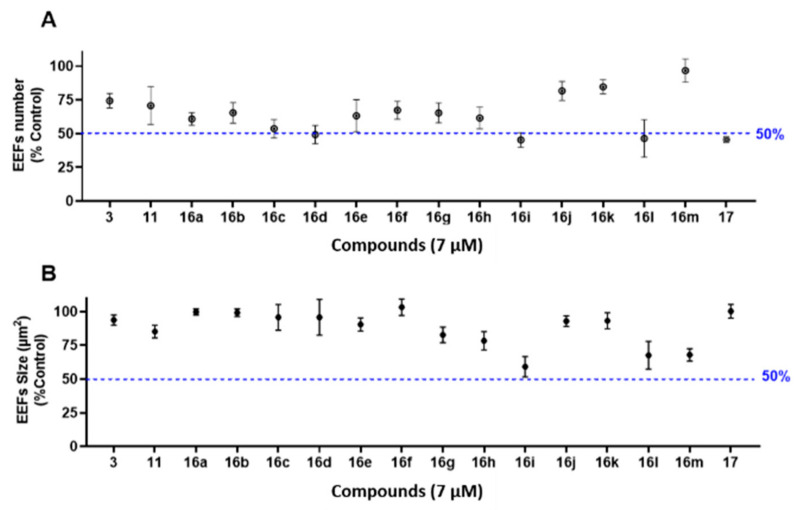
Normalized EEF number (**A**) and EEF size (**B**) in PHH-*Pb*-GFP at the arbitrary cut-off value of 7 μM for the indicated compounds. Each point (•) represents the average EEF number of four technical replicates. The error bars represent the interval of variability between EEF numbers. These results were obtained from four technical replicates.

The extent of DXM **3** metabolization in vivo into DX **12** (theoretically inhibited by QND) and to a lesser degree into *nor*-DXM **15** (3-methoxymorphinan) by CYP3A4-mediated *N*-demethylation [31,35] remains uncharacterized in our study. If effective and yielding inactive metabolites, its occurrence could explain the relatively poor prophylactic activity of DXM **3** in this mouse model of malaria. However, this outcome proved to be invalid, as both DX **12** and *nor*-DXM **15** were later synthesized (Figure 4) and found to have similar levels of inhibitory potency in vitro to DXM **3** against *P. berghei* (Figure 5 and Figure 6). Despite the unlikeliness of its repurposing as a prophylactic drug against malaria, DXM **3** represents a readily accessible and flexible synthetic platform upon which to explore the antimalarial potential of the *ent*-morphinan series, and circumvent the inherent limitations of the tazopsines.

### 3.3. DXM Pharmacomodulation towards Improved Antiplasmodial Derivatives

DXM **3**, possessing the same backbone as the natural hit tazopsine **1**, constituted the synthetic starting point of our study. This compound combines two advantages for a SAR study: (*i*) straightforward and cheap access from various commercial suppliers in gram quantities, and (*ii*) facile functionalization on the C-2 and/or *O*- and/or *N*-positions (Figure 4). To explore yet unraveled SARs on the aryl ring, DXM **3** was firstly *o*-iodinated using *N*-iodosuccinimide (NIS) to produce 2-I-DXM **11** with a 91% yield. DX **12** was obtained as described by Jakobsson et al. [17] by the *O*-demethylation of DXM **3** with 48% aqueous HBr, then *o*-iodinated with NIS under the previous conditions to produce 2-I-DX **13** with a 87% yield (Figure 4). Alkylation of the secondary amine position of tazopsine **1** constituting a relevant modification retaining the antiplasmodial activity of derivatives (Table 2) and possibly improving their antimalarial profile [14], *N*-modification of *nor*-DXM **15** was further explored. Towards this aim, DXM **3** free base was *N*-demethylated via the 2′,2′,2′-trichloroethylcarbamate intermediate **14**, as originally described by Peet et al. [18,20], to produce *nor*-DXM **15**. Reductive amination, particularly using sodium triacetoxyborohydride (STABH) as a reductant, represents a mild and chemoselective method for the *N*-alkylation of primary and secondary amines [36]. Tertiary amines **16a**–**m** were thus synthesized from *nor*-DXM **15** using *n*-alkyl, cycloalkyl, heteroaryl, and *bis*-heteroaryl aldehydes in presence of STABH, with moderate to high yields (33–99%) (Figure 4). To decipher the influence of the protonation state of the nitrogen atom on the antiplasmodial activity of *ent*-morphinans—taking into account that tazopsine **1**, *N*-cyclopentyl-tazopsine **2**, DXM **3**, DX **12**, *nor*-DXM **15**, and its *N*-alkyl derivatives **16a–m** are to be fully protonated at physiological pH values—two compounds **17** and **18** with a neutral or constitutively positive charge on the nitrogen atom, respectively, were synthesized. Cpd. **17**, corresponding to the exact amide congener of the amine **16d** for the purpose of precise SAR comparison, was prepared by reacting *nor*-DXM **15** with cyclopropanecarbonyl chloride in the presence of triethylamine, with a 75% yield. On the other hand, the quaternary ammonium **18** was obtained with a 82% yield by reacting DXM **3** free base with methyl iodide (Figure 4).

### 3.4. In Vitro Pre-Screening against P. berghei Liver Stages

The antiplasmodial activity against parasite liver stages of the obtained 21-compound Library was first pre-screened using PHH infected by *P. berghei* expressing the green fluorescent protein (PHH-*Pb*-GFP). This model has the advantage of being less expensive and more accessible than *P. falciparum* hepatic stages due to the routine production of *P. berghei* sporozoites in our laboratory, and also exploits the possible use of PHH to grow *P. berghei*, in order to be representative of *P. falciparum*–host cell interactions in a pre-screening context. Compound activity was assessed by two criteria: (*i*) the number of parasites developing within PHH (exoerythrocytic forms, EEFs, Figure 5A), and (*ii*) the size of parasites (μm^2^) normalized to untreated controls (Figure 5B). False positives, due to compound toxicity against host PHH, were excluded by normalizing the parasite number to the nuclei number of untreated controls. At the highest concentration (20 μM), viability of PHH was >80% for the least active compounds, and 50–60% for the most active compounds. Four activity profiles could be categorized from the 21-compound library pre-screening in terms of EEF number (Figure 5A), namely: (*i*) inactive compounds (2-I-DX **13**, **14**, **16f**, **16h**, **16j**, **17**, **18**); (*ii*) low-activity compounds with similar inhibitory potency to DXM **3** (DX **12**, *Nor*-DXM **15**, **16b**, **16c**, **16d**, **16e**, **16k**, and **16m**); (*iii*) active compounds (2-I-DXM **11**, **16a**, **16g**, and **16l**); (*iv*) one highly active compound (**16i**). These activity ranges were respectively characterized by: (*i*) a high EEF number at 20 μM (the maximal concentration of the range), (*ii*) a low EEF number at 20 μM, (*iii*) a low EEF number at 10 μM, and finally (*iv*) a low EEF number at 1 μM. We observed the same trend in the plot of parasite size (Figure 5B), with the delineation of cpd. **16i** exhibiting an important size effect at 1 μM, followed by cpds. **11**, **16c**, **16e**, **16g**, and **16l** at 10 μM. In the light of these results, we focused on compounds possessing an inhibitory activity between 1–10 μM, and established an amplified cut-off test at the arbitrary concentration of 7 μM to validate the above-described categories of inhibitors (Figure 6). This evaluation permitted to identify which compounds inhibited liver stage parasite development (e.g., leading to a 50% reduction in EEF number normalized to the drug-free controls). Five molecules were thus validated in the cut-off test, i.e., cpds. **16c**, **16d**, **16i**, **16l**, and **17** (Figure 6A). In addition, **16e** was intermediate and susceptible to exhibiting interesting activity. A low EEF size effect was observed for cpds. **16g**, **16h**, **16i**, **16l**, and **16m**, although none of these molecules inhibited parasite size by 50% or more (Figure 6B). Regarding the substitution of DXM **3** or DX **12** with iodine on the C-2 position, only 2-I-DXM **11** exhibited increased potency relative to the parent compound, whereas 2-I-DX **13** was inactive (Figure 5). Interestingly, amine **16d**, and its amide congener **17**, displayed similar levels of inhibitory activity against *P. berghei* liver stages (Figure 5 and Figure 6). This suggested that the presence of an electrodonating doublet on the nitrogen atom, rather than that of a protonated doublet, was an important feature for the interaction of *ent*-morphinans with their biochemical target(s). However, this behavior was contradictory to that observed in the tazopsine series, whereby *N*-acetylation abolished activity (Table 2). Nevertheless, this trend in the DXM series was reinforced by the lower activity of the quaternary ammonium **18** compared to DXM **3** (Figure 5). This showed that, despite the intrinsically charged state of most bioactive DXM derivatives at physiological pH (with the exception of cpd. **17**), the presence of a permanently charged nitrogen atom was detrimental to the antiplasmodial activity.

### 3.5. In Vitro Screening against P. falciparum Liver and Asexual Blood Stages

2-I-DXM **11**, **16a** (R = *N*-*n*-propyl), **16g** (R = *N*-2′-furanylmethyl), **16i** (R = *N*-2${}^{\prime }$-pyrrolylmethyl), and **16l** (R = *N*-2${}^{\prime }$-indolylmethyl) displayed the best inhibitory effects in the *P. berghei* pre-screening and were thus selected for screening against both the liver and blood stages of the human parasite *P. falciparum* and the determination of their IC_50_ values. PQ and chloroquine (CQ) were used as reference drugs against the liver and blood stages, respectively. Compound toxicity against host PHH was excluded by normalizing the parasite number to the nuclei number of untreated controls. At 10 μM, the viability of PHH was ca. 100% for the least active compounds, and 60–70% for the most active compounds. The observed increase in the antiplasmodial activity of 2-I-DXM **11** compared to DXM **3** against *P. berghei* liver stages was confirmed against *P. falciparum*, the latter being 4-fold more active than the parent compound (Table 4). Regarding the pre-screened *N*-modified analogues **16a**, **16g**, **16i**, and **16l**, these were active against *P. falciparum* liver stages in the low micromolar/submicromolar range and displayed significantly lower IC_50_ values than both parent compounds, i.e., the natural hit tazopsine **1** and DXM **3** (Table 2 and Table 4). The most active compound was the *N*-2${}^{\prime }$-pyrrolylmethyl derivative **16i**, which strongly inhibited the development of parasite liver stages, being 10-fold more active than tazopsine **1** (Table 2) and 20-fold more active than DXM **3** (Table 4). Strikingly, cpd. **16i** showed a superimposable inhibitory potency to the antimalarial drug PQ against *P. falciparum* liver stages (IC_50_ values of 0.76 ± 0.13 μM and 0.75 ± 0.15 μM, respectively). Cpd. **16i** was also very active on the parasite blood stages (IC_50_ values of 2.1 ± 0.4 μM), again similarly to PQ, which has reported IC_50_ values against the *P. falciparum* 3D7 strain in the 1–20 μM IC_50_ range [24,37]. With the exception of **16i**, only **16l** showed activity in the low micromolar range against *P. falciparum* blood stages (IC_50_ of 6.5 ± 0.4 μM). Other *N*-alkylated derivatives exhibited low activity against blood stages, similar to DXM **3**, with IC_50_ values of 43–62 μM. However, all compounds were found to be selective for the hepatic phase of parasite development (2.8–36-fold selectivity) including DXM **3** (4.9-fold selectivity) (Table 4).

### 3.6. In Vitro Screening against P. falciparum Sexual Blood Stages

**Figure 7:**
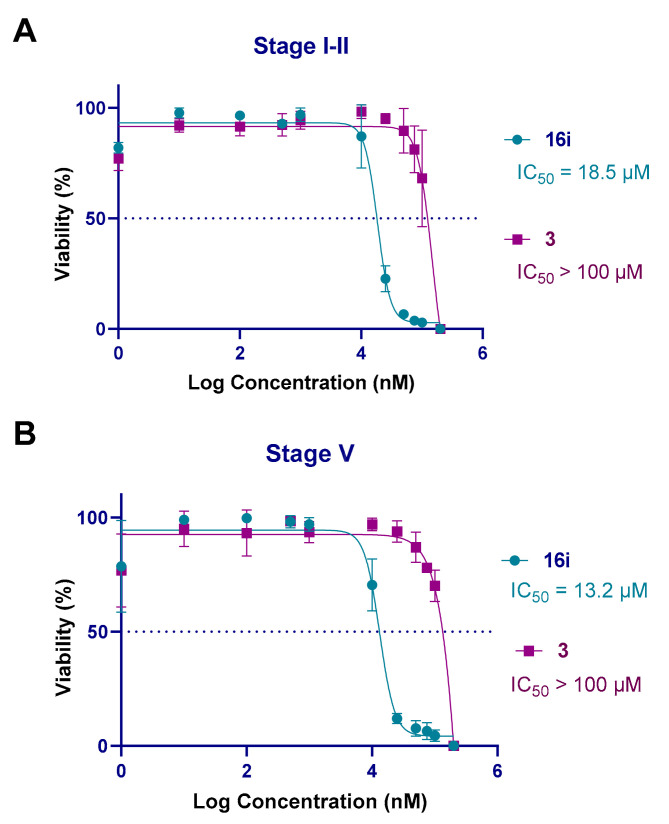
Inhibitory plots and IC_50_ values of DXM **3** (purple) and cpd. **16i** (cyan) against *P. falciparum* early (stages I–II) (**A**) and late (stage V) (**B**) gametocytes in vitro. IC_50_ values are the mean of three technical replicates and three biological replicates. Error bars represent standard deviation.

To explore the activity profile of the new hit cpd. **16i** against other developmental stages of *P. falciparum*, we tested it against early (stages I–II) and late (stage V) gametocytes, the last being responsible for the transmission of *P. falciparum* malaria. Cpd. **16i** was found to be fairly effective against both gametocyte stages, with activities in the high micromolar range (IC_50_ values of 18.5 μM and 13.2 μM, respectively), whereas DXM **3** was completely inactive (IC_50_ > 100 μM) (Figure 7).

## 4. Conclusions

The rapid spread of artemisinin-resistant *P. falciparum* strains across Southeast Asia [5] and independent increase of k13 polymorphisms in Africa [38,39], which culminated in clinical artemisinin resistance recently being detected in the continent [6,7,8], is a dramatic continuum of the drug resistance history of malarial parasites. This situation underscores the need for alternative chemotherapeutic strategies beyond pursuing novel drugs to eliminate blood parasites, prone to acquiring resistance mutations. In this context, the antiplasmodial alkaloid tazopsine **1**, introduced in the late 2000s, is active against both pre-erythrocytic and erythrocytic *P. falciparum* stages, and constitutes the precursor of the in vivo prophylactic compound *N*-cyclopentyl-tazopsine **2**. However, the development of the tazopsines through biosourcing or synthetic strategies was difficult. Aiming to overcome these limitations, we managed to simplify the natural alkaloid tazopsine **1**—the same principle also applying to the related sinococuline **5**—and to extract a “naked” *ent*-morphinan antiplasmodial pharmacophore in the form of the antitussive drug DXM **3**. In particular, substitutions at the level of rings B and C proved non-essential, whereas those affecting ring A (particularly at C-2) were of potential relevance. Despite its limited potential for a repurposing against malaria, DXM **3** exhibited a significant level of bioactivity in vitro, that was only 2-fold lower than that of tazopsine **1** against *P. falciparum* liver stages. Capitalizing on strengthened ring D SARs, a rapid diversification of DXM **3** into *N*-modified derivatives readily led to improved analogues. Amongst those, the hit cpd. *N*-2${}^{\prime }$-pyrrolylmethyl-*nor*-DXM **16i** exhibited a 10-fold and 20-fold increase in activity against *P. falciparum* liver stages relative to tazopsine **1** and DXM **3**, respectively, and showed similar activity than the reference drug primaquine against parasite liver and blood stages. In the context of prophylactic antimalarial drug discovery, cpd. **16i** was more selective for the parasite liver phase than tazopsine **1** (S = 2.8 vs. 0.5) in addition to having a stronger bioactivity than the natural alkaloid. Moreover, cpd. **16i** had a significant effect on the viability of stage I-II and V *P. falciparum* gametocytes compared to the inactive DXM **3**. These results warrant further mechanistic investigation into the new hit cpd. **16i** regarding causal prophylaxis (i.e., early sterilization of sporozoites) and possible pan-activity against multiple parasite stages. In addition, the benefit of C-2 substitution suggests it may be possible to further optimize derivatives in the hit series by means of ring A modifications.

## Figures and Tables

**Table 1:** Reaction times to synthesize cpds. **16a**–**16m** by reductive amination with STABH, and their yields.

Cpd.	Reaction Time (h)	Yield (%)
**16a**	2	45
**16b**	2	75
**16c**	2	56
**16d**	2	56
**16e**	2	52
**16f**	2	68
**16g**	2	35
**16h**	2	68
**16i**	2	53
**16j**	21	48
**16k**	15	33
**16l**	2	82
**16m**	2	97

**Table 2:** In vitro IC_50_ values of tazopsine **1**, derivatives **6**–**8** and natural *ent*-morphinan alkaloids in *P. yoelii* liver stages. IC_50_ values are the mean of four technical replicates. PMH: primary mouse hepatocytes; NA: not applicable; PQ: primaquine.

Cpd.	Substitutions	IC_50_ (Py265BY-PMH, µM)
Tazopsine **1**	R_1_ = OH, R_2_ = H	3.1 ± 0.2
10-*epi*-tazopsine **4**	R_1_ = H, R_2_ = OH	16.1 ± 1.9
Sinococuline **5**	R_1_ = R_2_ = H	4.5 ± 0.4
4-*O*-Me-tazopsine **6**	NA	>100
*N*-methyl-tazopsine **7a**	R = Me	5.8 ± 0.4
*N*-*n*-propyl-tazopsine **7b**	R = *n*-Pro	12.6 ± 1.7
*N*-4′-hydroxybenzyl-tazopsine **7c**	R = 4-OH-Bn	14.2 ± 2.2
*N*-4′-methoxybenzyl-tazopsine **7d**	R = 4-OMe-Bn	24.2 ± 0.7
*N*-3′,4′-methylenedioxybenzyl-tazopsine **7e**	R = 3,4-methylenedioxy-Bn	>100
*N*-4′-chlorobenzyl-tazopsine **7f**	R = 4-Cl-Bn	5.8 ± 1.1
*N*-4′-bromobenzyl-tazopsine **7g**	R = 4-Br-Bn	4.2 ± 0.3
*N*-acetyl-tazopsine **8**	R = Ac	>100
Sinoacutine **9**	NA	>100
Sinomenine **10**	NA	>100
PQ	NA	0.62 ± 0.03

**Table 3:** In vitro IC_50_ values of tazopsine **1** and DXM **3** in *P. falciparum* liver stages. IC_50_ values are the mean of four technical replicates. PHH, primary human hepatocytes; PQ, primaquine.

Cpds.	IC_50_ (PHH, µM)
Tazopsine **1**	7.88 ± 3.05
DXM **3**	15.59 ± 1.19
PQ	0.75 ± 0.15

**Table 4:** In vitro IC_50_ values of tazopsine **1**, DXM **3**, and selected *N*-modified derivatives against *P. falciparum* liver (NF54) and blood (3D7) stages, as well as liver phase selectivity values (S). IC_50_ values are the mean of four technical replicates for PHH and three technical replicates for HE. R identities for *N*-substituted DXM **3** derivatives refer to Figure 4. HE: human erythrocytes; NT: not tested; NA: not applicable.

Cpds.	R	IC_50_ (µM)		S
		PfNF54-PHH	Pf3D7-HE	
Tazopsine **1**	NA	7.88 ± 3.05	4.07 ± 0.87	0.5
DXM **3**	Me	15.59 ± 1.19	76.5 ± 0.9	4.9
**11**	NA	4.10 ± 2.77	61.7 ± 5.3	15.0
**16a**	*n*-propyl	2.25 ± 3.03	43.3 ± 2.3	19.2
**16g**	2′-furanylmethyl	1.56 ± 0.59	56.2 ± 2.7	36.0
**16i**	2′-pyrrolylmethyl	0.76 ± 0.13	2.1 ± 0.4	2.8
**16l**	2′-indolylmethyl	1.98 ± 0.34	6.5 ± 0.4	3.3
PQ	NA	0.75 ± 0.15	NT	NA
CQ	NA	NT	0.033 ± 0.016	NA

## Data Availability

Not applicable.
